# FGF21 induced by endoplasmic reticulum stress maintains medullary thymic epithelial cell function and central immune tolerance

**DOI:** 10.1126/sciadv.aee9999

**Published:** 2026-07-17

**Authors:** Yuki Masuda, Yoshiaki Nakayama, Ryohei Shimizu, Hiroshi Hasegawa, Morichika Konishi

**Affiliations:** ^1^ Laboratory of Microbial Chemistry, Kobe Pharmaceutical University; Kobe 658-8558, Japan.; ^2^ Department of Molecular Pharmaceutics, Hoshi University; Tokyo 142-8501, Japan.; ^3^ Laboratory of Hygienic Sciences, Kobe Pharmaceutical University; Kobe 658-8558, Japan.

## Abstract

Medullary thymic epithelial cells (mTECs) establish central immune tolerance by expressing diverse tissue-restricted antigens and eliminating self-reactive thymocytes. Here, we show that fibroblast growth factor 21 (FGF21), although predominantly produced in the liver, is also expressed locally by mature mTECs and contributes to central tolerance. *Fgf21*-deficient mice exhibited exacerbated peripheral autoimmune responses. FGF21 supported the number and function of mTECs and promoted clonal deletion in cooperation with thymic dendritic cells. In mature mTECs, endoplasmic reticulum stress induced FGF21 expression through unfolded protein response pathways, with FGF21 acting preferentially within the mature mTEC compartment as a stress-responsive metabolic regulator downstream of the integrated stress response. By limiting sustained stress and preserving protein homeostasis, FGF21 maintained mTEC integrity and central tolerance. These findings identify FGF21 as a key regulator of thymic immune homeostasis and as a potential therapeutic target for autoimmune disease.

## Teaser

FGF21 induced by endoplasmic reticulum stress in mTECs preserves protein homeostasis and supports central immune tolerance.

## INTRODUCTION

Central immune tolerance is a fundamental process by which developing T cells are educated within the thymus to differentiate between self and non-self components (*1*, *2*). During thymocyte differentiation, αβ T cells undergo a stepwise selection process that includes both positive and negative selection (*3*). In the cortex, only CD4^+^CD8^+^ double-positive (DP) thymocytes with low-affinity recognition of MHC/self-peptide complexes (pMHC) presented by cortical thymic epithelial cells (cTECs) receive the survival and differentiation signals necessary to progress through positive selection. Subsequently, positively selected DP thymocytes that strongly react to pMHC presented primarily by cortical dendritic cells (DCs) are eliminated during the early phase of negative selection (*4*). DP cells that escape this checkpoint differentiate into CD4 or CD8 single-positive (SP) cells, upregulate CCR7, and migrate from the cortex to the medulla.

Within the medulla, thymocytes undergo the later phase of negative selection, a process driven by strong interactions with pMHC mainly presented by medullary thymic epithelial cells (mTECs) and thymic DCs (*5*–*8*). mTECs play a central role by expressing a diverse repertoire of tissue-restricted antigens (TRAs), largely under the control of the transcription factor autoimmune regulator (Aire), and presenting these self-antigens to developing thymocytes (*3*, *9*). Thymic DCs also acquire and present self-antigens derived from mTECs (*10*). Together, mTECs and DCs form a specialized medullary microenvironment that ensures the deletion of strongly self-reactive clones or their diversion into the regulatory T cell (T_reg_) lineage. Because disruption of these tolerance mechanisms leads to autoimmune disease, elucidating the pathways that sustain mTEC and DC function is critically important.

Fibroblast growth factor 21 (FGF21) is an endocrine factor mainly secreted by the liver that regulates glucose and lipid metabolism (*11*). The therapeutic potential of FGF21 for metabolic disorders is under active investigation (*12*). We previously showed that *Fgf21* is highly expressed in mature mTECs (*13*), whereas other groups showed that its overexpression in TECs protects against age-related thymic atrophy by maintaining TEC numbers (*14*, *15*). *Fgf21* expression in the thymus begins around embryonic day 15.5 and peaks at 1–2 months, coinciding with maximal thymic function (*13*). However, the physiological role of constitutive thymic FGF21 remains largely unknown. Our previous work revealed an increased proportion of immature T cells in *Fgf21* knockout (*Fgf21^−/−^*) mice and that recombinant FGF21 induced apoptosis of immature T cells in fetal thymus organ culture (FTOC) (*13*). These findings suggest that FGF21 contributes to thymic T cell selection. In this study, we examined the physiological role of FGF21 in maintaining central immune tolerance.

## RESULTS

### Deficiency of *Fgf21* increases autoimmune responses via the thymus

**Fig. 1.**
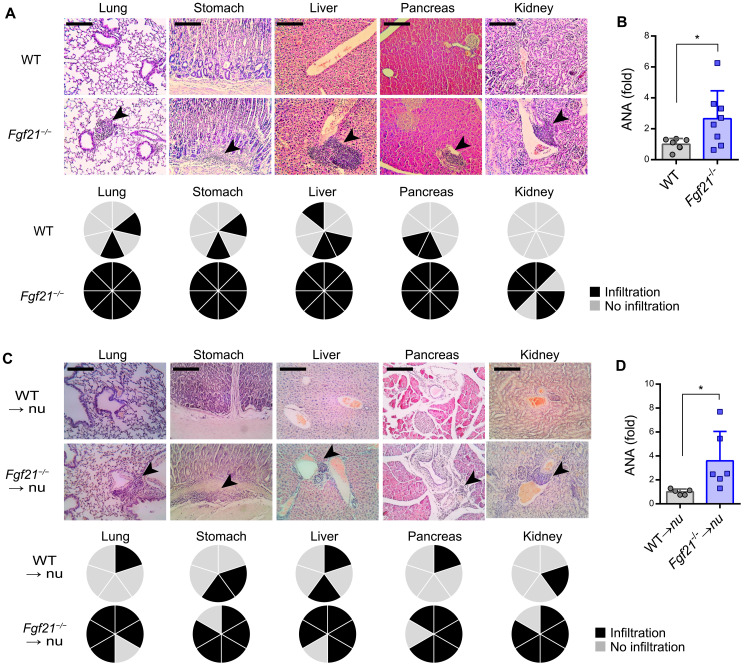
*Fgf21* deficiency induces an autoimmune response. **A**) Representative hematoxylin and eosin (H&E)-stained images of the lungs, stomach, liver, pancreas, and kidneys from 14-month-old WT (*n* = 7) and *Fgf21^−/−^* (*n* = 8) mice. (**B**) Serum ANA levels are shown as relative amounts compared with WT. (**C**) H&E images of nude mice transplanted with WT or *Fgf21^−/−^* thymi (WT → nu, *n* = 5; *Fgf21^−/−^* → nu, *n* = 6). (**D**) Serum ANA levels in transplanted mice. Lymphocytic infiltration (arrowheads) and elevated ANA indicate autoimmune activation. Scale bar, 200 μm. Data are presented as mean + SD. Two-tailed unpaired Student’s *t* tests were used for statistical analysis. *P < 0.05. Each experiment was independently repeated at least twice with similar results.

Wedemeyer *et al*. reported that mTEC-specific overexpression of *Fgf21* reduces peripheral autoimmune responses in 14-month-old middle-aged mice (*14*). To test whether physiological FGF21 is required to suppress autoimmunity, we examined lymphocyte infiltration—a marker of autoimmune responses—in the lungs, stomach, liver, pancreas, and kidneys of *Fgf21^−/−^* and wild-type (WT) mice. Although no lymphocytic infiltration was observed in 2-month-old *Fgf21^−/−^* and WT mice (fig. S1), clear infiltration was observed in independently examined 14 months of age (Fig. 1A), consistent with a previous report that age-associated autoimmunity increases with aging (*16*). Lymphocyte infiltration was more frequent in *Fgf21^−/−^* than in WT mice (Fig. 1A), accompanied by higher serum antinuclear antibody (ANA) levels (Fig. 1B), indicating that physiological deficiency of *Fgf21* increases susceptibility to autoimmunity, consistent with impaired central tolerance.

We then explored whether the autoimmune responses observed in *Fgf21^−/−^* mice were linked to thymic immune tolerance. The genetic background of mouse strains significantly affects the severity of autoimmunity, with the C57BL/6 background exhibiting only mild symptoms (*17*). This genetic factor may explain why autoimmune responses were absent in 2-month-old *Fgf21^−/−^* mice with a C57BL/6 background (fig. S1). Following a previous study (*18*), we performed allogenic thymus-transplantation experiments. Fetal thymi from E15.5 *Fgf21^−/−^* or WT embryos were treated with 2-deoxyguanosine (2DG) to remove lymphocytes and transplanted under the kidney capsule of BALB/c nude mice. Ten weeks after transplantation, recipients of *Fgf21^−/−^* thymi (*Fgf21^−/−^* → nu) displayed more lymphocytic infiltration (Fig. 1C) and higher serum ANA levels than those of WT thymi (WT → nu) (Fig. 1D). These results indicate that thymic physiological FGF21 is required to maintain peripheral immune tolerance.

### Deficiency of *Fgf21* exacerbates experimental autoimmune encephalomyelitis

**Fig. 2.**
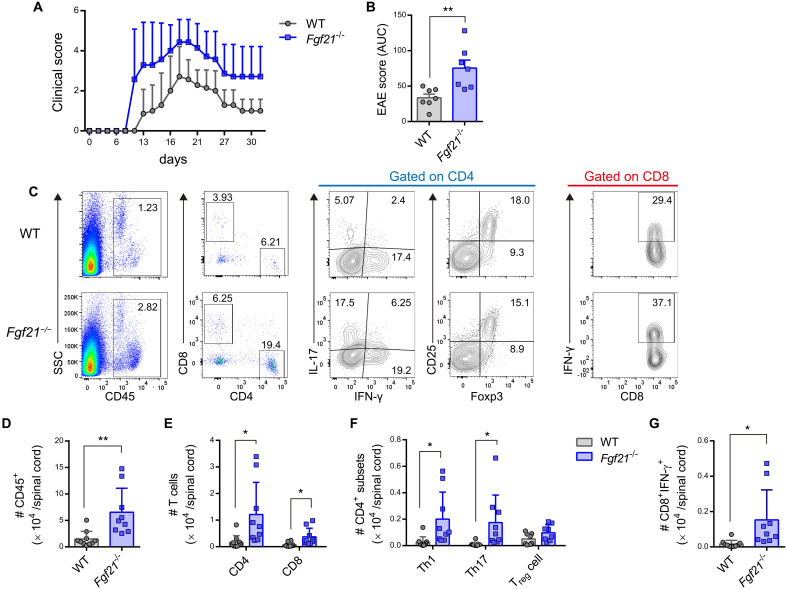
*Fgf21* deficiency exacerbates EAE severity. Changes in clinical scores (**A**) and AUC (**B**) after EAE induction in 2-month-old WT and *Fgf21^−/−^* mice are shown (*n* = 7). (**C**) Representative results of flow cytometry analysis of the spinal cord on day 18 after EAE. The numbers of CD45^+^ immune cells (**D**), CD4^+^ T cells, CD8^+^ T cells (**E**), Th1 or Th17, T_reg_ cells **(F)**, and IFN-γ^+^ CD8^+^ T cells (**G**) are shown (*n* = 8–10). For statistical analysis, Mann–Whitney U test (B), and two-tailed unpaired Student’s *t* tests [(D) to (G)] were used. *P < 0.05 and **P < 0.01.

To evaluate the role of FGF21 in autoimmune disease, we used experimental autoimmune encephalomyelitis (EAE), a T cell–dependent model of multiple sclerosis. EAE is initiated by peripheral priming of T cells with the myelin oligodendrocyte glycoprotein (MOG)_35−55_ peptide, a central nervous system (CNS)-specific self-antigen, followed by their migration into the CNS (*19*). After MOG_35–55_ immunization, clinical symptoms appeared on day 12 in 2-month-old WT mice, whereas *Fgf21^−/−^* mice tended to show higher clinical scores throughout the observation period (Fig. 2A), analysis of the area under the curve (AUC) demonstrated a significantly greater overall disease burden compared with WT mice (Fig. 2B).

Flow cytometry revealed increased frequencies of CD45^+^, CD4^+^, and CD8^+^ T cells in the spinal cords of *Fgf21^−/−^* mice (Fig. 2, C to E). Because Th1 and Th17 effector CD4^+^ subsets drive EAE (*19*), we quantified IFN-γ^+^ (Th1) and IL-17A^+^ (Th17) CD4^+^T cells. Both populations were significantly elevated in *Fgf21^−/−^* mice (Fig. 2, C and F). IFN-γ–producing CD8^+^ T cells were also increased, whereas Foxp3^+^ T_reg_ cell numbers were unchanged (Fig. 2, C, F, and G). These results indicate that *Fgf21* loss exacerbates EAE, accompanied by enhanced generation of autoreactive effector T cells.

### *Fgf21* deficiency impairs clonal deletion but not T_reg_ cell generation

At 1 month of age, total thymocyte numbers were slightly but significantly higher in *Fgf21^−/−^* than in WT mice (fig. S2A). The number of CD4^−^CD8^−^-double-negative (DN), CD4 SP, and CD8 SP cells was unchanged, whereas DP thymocytes were significantly increased in *Fgf21^−/−^*mice (fig. S2B), suggesting altered T cell selection.

**Fig. 3.**
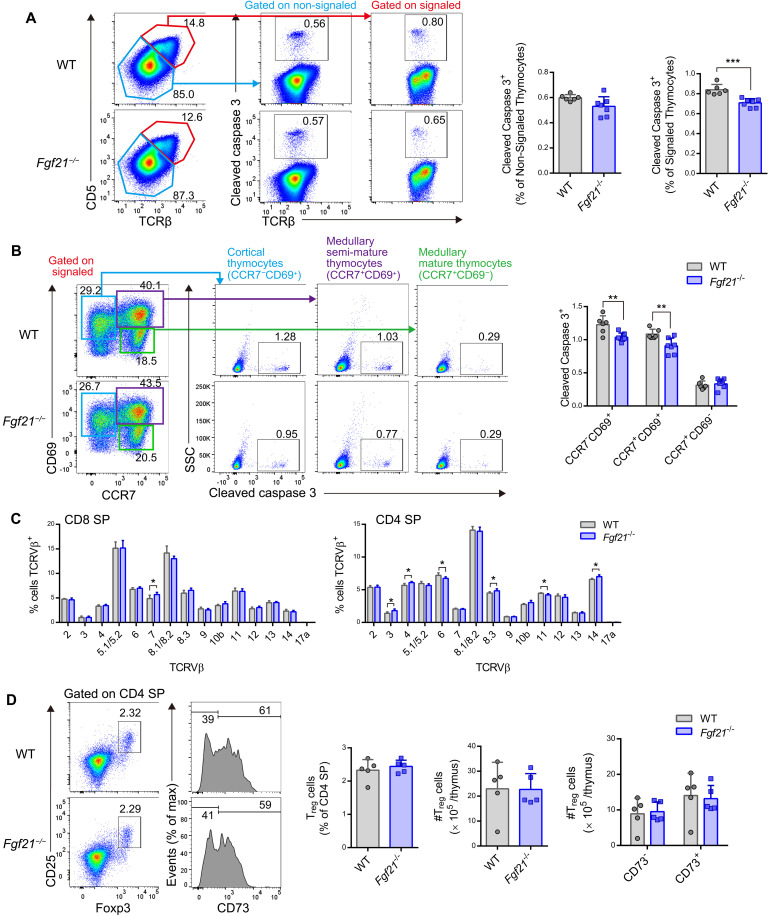
*Fgf21* deficiency impairs thymic clonal deletion. (**A** and **B**) Flow cytometric identification of death by neglect and clonal deletion thymocytes in 1-month-old WT and *Fgf21^−/−^* mice (*n* = 6–7). (**C**) TCRVβ distribution in CD8 SP and CD4 SP was analyzed by flow cytometry. (**D**) Frequency of T_reg_ cells in CD4 SP, number of T_reg_ cells in the thymus, number of newly generated thymic CD73^−^ T_reg_ cells, and number of recirculating CD73^+^ T_reg_ cells (*n* = 5). Two-tailed unpaired Student’s *t* tests were used for statistical analysis. *P < 0.05, **P < 0.01, and ***P < 0.001.

Clonal deletion can be analyzed using a flow cytometry-based approach (*7*). Among CD5^−^TCRβ^−^ non-signaled thymocytes, cells that do not receive productive TCR signals during positive selection undergo apoptosis (death by neglect). When T cell receptor signaling is induced during positive selection, these cells upregulate CD5 and TCRβ and become CD5^+^TCRβ^+^-signaled thymocytes. Among these, cells with excessive self-reactivity undergo apoptosis (clonal deletion). In the *Fgf21^−/−^* thymus, there was no change in the frequency of death by neglect, but the frequency of clonal deletion was significantly decreased compared to that in the WT (Fig. 3A). These findings align with a previous study (*14*) that demonstrated an increase in clonal deletion in 2-month-old mice with *Fgf21* overexpression in mTECs. Among the signaled thymocytes, the frequency of cleaved caspase3^+^ apoptotic cells in cortical thymocytes (CCR7^−^CD69^+^) and medullary semi-mature thymocytes (CCR7^+^CD69^+^) was significantly reduced in *Fgf21^−/−^* mice, whereas no change was observed in medullary mature thymocytes (CCR7^+^CD69^−^) (Fig. 3B). These results suggest that physiological *Fgf21* deficiency compromises clonal deletion at both the cortical and medullary stages. Consistently, TCRVβ repertoire analysis revealed subtle but significant alterations in CD4 SP and CD8 SP thymocytes from *Fgf21^−/−^* mice compared with those from WT (Fig. 3C).

In addition to clonal deletion, T_reg_ cell generation is essential for central tolerance, and the thymic medulla provides the microenvironment for T_reg_ cell differentiation (*20*). The proportion and total number of Foxp3^+^ T_reg_ cells were comparable between *Fgf21^−/−^* and WT thymi (Fig. 3D). As nearly half of thymic T_reg_ cells in 1-month-old mice are CD73^+^ cells recirculating from the periphery (*21*, *22*), we analyzed both subsets and found no differences in newly generated CD73^−^ or recirculating CD73^+^ T_reg_ cells (Fig. 3D). Consistent with the unchanged number of CNS-infiltrating T_reg_ cells in EAE, these data indicated that *Fgf21* deficiency did not substantially alter the overall number of thymic T_reg_ cells. However, because the TCR repertoire and other qualitative features of T_reg_ cells were not examined in this study, the possible contribution of altered T_reg_ cell selection cannot be excluded. In mTEC-specific *Fgf21*-overexpressing mice, the number of thymic T_reg_ cells was unaffected at 2 months but increased at 12 months (*14*), suggesting that FGF21 may indirectly modulate thymic T_reg_ cell output through age-related changes in the thymic microenvironment.

To assess peripheral consequences of thymic changes, we examined the spleen. Total splenocyte numbers in 1-month-old *Fgf21^−/−^* mice were similar to those in WT (fig. S3A). Although the frequency of CD4 T and CD8 T cells was slightly lower, the absolute numbers of CD4 T and CD8 T cells were unchanged (fig. S3B). A reduction in naïve T cells in the spleen is characteristic of age-related thymic involution, and in aged *Fgf21*-overexpressing mice, this decline was alleviated (*14*, *15*). In the spleens of 1-month-old *Fgf21^−/−^* mice, the numbers of naïve or effector memory CD4 T or CD8 T cells showed no differences compared to those in WT mice (fig. S3C). Similar to the thymus, in the spleen, *Fgf21* deficiency did not affect the number of T_reg_ cells in the spleen (fig. S3D).

### *Fgf21* deficiency reduces the proliferation and survival of mTECs

**Fig. 4.**
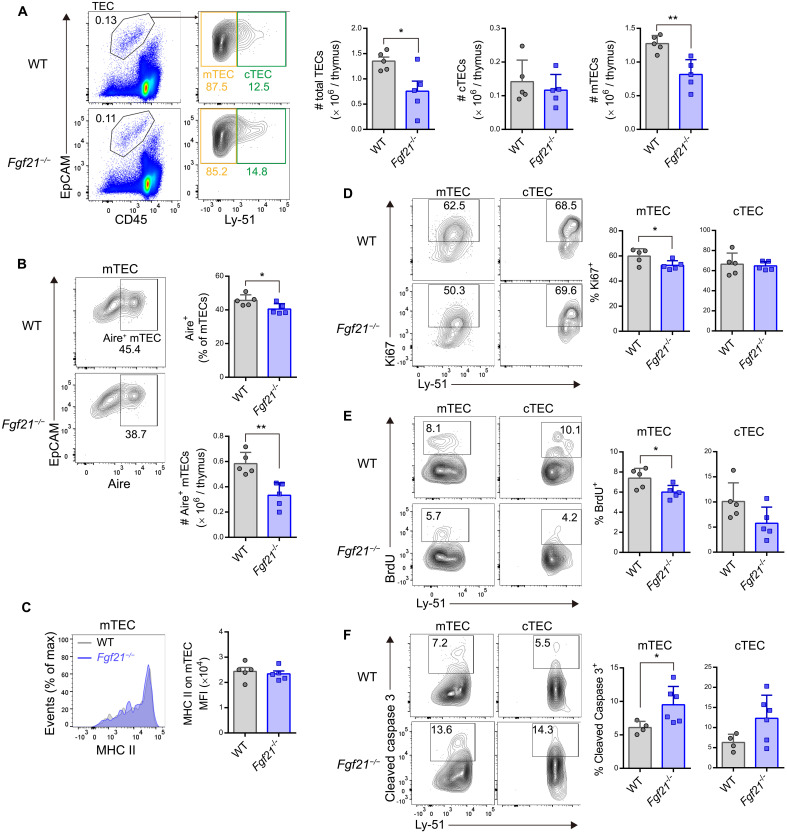
*Fgf21* deficiency reduced the number of mTECs. (**A** to **D**) Cell numbers of TECs, mTECs, cTECs, Aire^+^ mTECs, and MHC class II expression on mTECs from 1-month-old WT and *Fgf21^−/−^* mice (*n* = 5). (**D** to **F**) Ki67^+^, BrdU^+^, and cleaved caspase 3^+^ of mTECs/cTECs (*n* = 5). Two-tailed unpaired Student’s *t* tests were used for statistical analysis. *P < 0.05 and **P < 0.01.

Although *Fgf21* overexpression has been reported to prevent thymic involution by preserving mTEC and cTECs numbers in aged mice, findings regarding its role in the young thymus have been inconsistent and remain unclear (*14*, *15*). Within the thymus of 1-month-old *Fgf21^−/−^* mice, cTEC numbers were unchanged, but mTECs were markedly reduced, accompanied by fewer Aire^+^ mTECs (Fig. 4, A and B). These results indicate that physiological FGF21 is required to maintain mature mTECs in the young thymus. In contrast, no difference was noted in the expression levels of MHC class II molecules in the mTECs (Fig. 4C). As previously reported, the overall cortical and medullary organization of the thymus is grossly preserved in young *Fgf21^−/−^* mice (*13*), suggesting that the reduction in the number of mTECs reflects decreased cellularity rather than overt disruption of the medullary architecture.

Ki67 staining and BrdU incorporation revealed reduced mTEC proliferation in *Fgf21^−/−^* mice (Fig. 4, D and E), whereas the frequency of cleaved caspase-3^+^ apoptotic cells was increased (Fig. 4F). cTECs showed a similar but nonsignificant trend (Fig. 4, E and F). These observations indicate that physiological FGF21 promotes mTEC proliferation and survival.

### *Fgf21* deficiency reduces pMHC transfer from mTECs to thymic cDCs

**Fig. 5.**
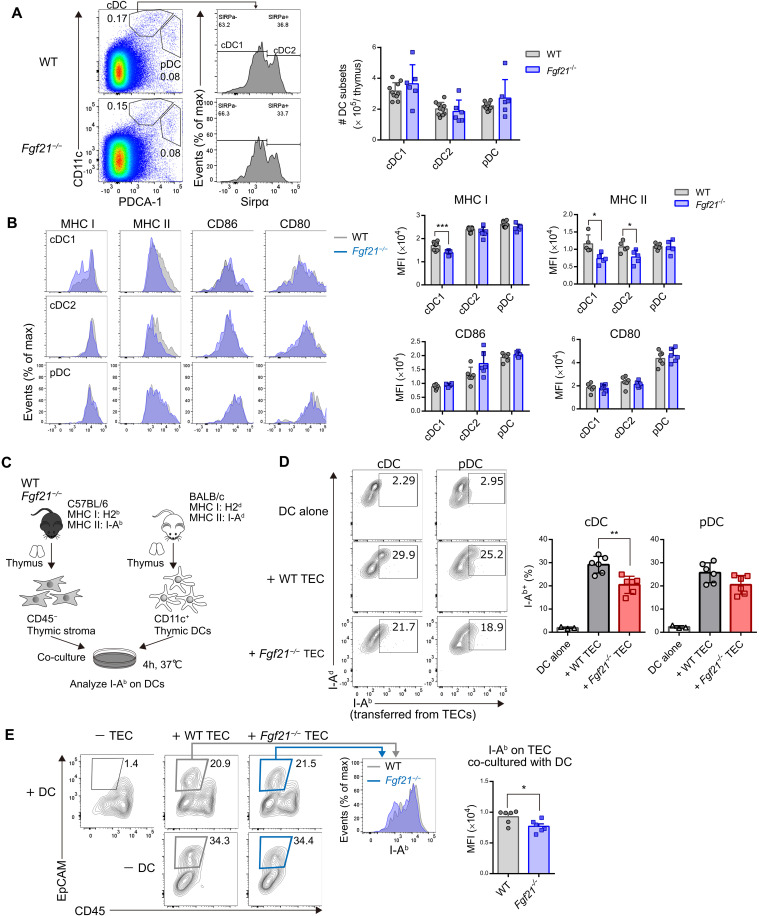
*Fgf21* deficiency decreases MHC expression in thymic DC. (**A**) Thymic DC subsets (cDC1, cDC2, pDC) and cell numbers (*n* = 6–10). (**B**) Expression of MHC class I/II and costimulatory molecules (*n* = 5–6). (**C** to **E**) In vitro MHC-transfer assays between TECs and DCs using MHC-mismatched strains. For statistical analysis, two-tailed unpaired Student’s *t* tests [(A), (B), and (E)] and one-way ANOVA followed by Tukey’s post-hoc test (D) were used. *P < 0.05, **P < 0.01, and ***P < 0.001.

The thymus contains three DC subsets: plasmacytoid DCs (pDCs), classical DC1 (cDC1), and DC2 (cDC2) (*23*). Cooperative antigen transfer (CAT) from mTECs to thymic cDCs, enabling presentation of mTEC-derived pMHC to thymocytes, is essential for central tolerance (*23*, *24*). In 1-month-old *Fgf21^−/−^* mice, total numbers of cDC1, cDC2, and pDCs were comparable to those in WT (Fig. 5A), but MHC class I/II expression was reduced in cDC1 and class II in cDC2 (Fig. 5B). In contrast, the expression levels of the costimulatory factors CD86 and CD80 were unchanged (Fig. 5B). No changes were observed in the mRNA expression of I-Aα (*H2-Aa*) and I-Aβ (*H2-Ab1*) in *Fgf21^−/−^* thymus-derived CD11c^+^ DCs (fig. S4A), suggesting that FGF21 affects MHC protein levels on the surface of thymic cDCs. No differences were seen in MHC II expression on splenic DC subsets (fig. S4, B and C). These results suggest that FGF21 specifically influences the antigen-presenting function of cDCs in the thymus.

FGF21 signals through FGFR1 in complex with the co-receptor KLB (*25*). *Klb* expression was detected in mTECs but not in T cell lineages, fibroblasts, or macrophages (*14*). Consistent with this previous report, *Klb* expression was detected in mTEC but not in any DC subset or in cTEC (fig. S5A). Mature mTEC^hi^ (MHC II^hi^ CD45^−^EpCAM^+^) showed higher *Klb* expression than immature mTEC^lo^ (MHC II^lo^ CD45^−^EpCAM^+^) (fig. S5A). *Fgfr1* expression was detected in all mTEC, cTEC, and DC subsets, with particularly high expression in cDC1 and pDC (fig. S5A). When FGF21 binds to KLB and FGFR, it induces intracellular signaling and triggers extracellular signal-regulated kinase (ERK) phosphorylation (*25*). The addition of recombinant FGF21 (rFGF21) protein to thymic single-cell suspensions induced ERK phosphorylation in mTEC but not in cTEC, DC subsets, CD45^+^ thymocytes, or CD45^−^EpCAM^−^ cells (fig. S5B), indicating that FGF21 acts directly on mTECs.

CAT involves either phagocytosis of apoptotic mTECs by cDCs or direct membrane transfer, known as “dendritic cell cross-dressing,” which involves intercellular transfer of cell membrane components, including pMHC, from mTECs to cDCs (*26*–*28*). Using MHC-mismatched co-culture, we assessed pMHC transfer from mTECs to DCs (*26*). Stromal cells (CD45^−^), including mTECs from WT or *Fgf21^−/−^* (C57BL/6, H2^b^) mice were co-cultured 4 hours with BALB/c DCs (H2^d^). The frequency of I-A^b+^ cells in cDCs and pDCs when co-cultured with TECs increased relative to DC-only cultures, confirming pMHC transfer (Fig. 5D). However, I-A^b+^ cDCs were significantly fewer when co-cultured with *Fgf21^−/−^* TECs, and a similar trend was observed in pDCs, although the difference was not statistically significant (Fig. 5D). Although mTEC MHC II expression in vivo was unchanged (Fig. 4C), I-A^b^ levels declined in *Fgf21^−/−^* TECs after culture (Fig. 5E), suggesting reduced stability of MHC molecules and less efficient pMHC transfer to DCs.

### Aged *Fgf21*-deficient mice retain reduced mTEC populations at the onset of autoimmunity

Because autoimmune phenotypes became evident only in aged *Fgf21^−/−^* mice, we examined thymic stromal populations in 14-month-old mice. At this age, thymus weight and total thymocyte number were comparable between WT and *Fgf21^−/−^* mice, indicating no gross difference in overall thymic size or cellularity (fig. S6A). Nevertheless, aged *Fgf21^−/−^* mice showed a reduced mTEC compartment (fig. S6, B and C), consistent with the defect observed in young mice. Although the overall composition of thymic DC subsets was largely unchanged, MHC II expression remained modestly reduced (fig. S6, D and E). Because central tolerance depends on both mTEC abundance and CAT from mTECs to DCs, these findings raise the possibility that persistent reduction of the mTEC compartment, together with impaired mTEC-to-DC antigen transfer, contributes to the autoimmune manifestations that emerge in aged *Fgf21*-deficient mice.

### FGF21 enhances clonal deletion, Aire^+^ mTECs, and MHC expression on cDC1

To determine whether secreted FGF21 acts directly within the thymus and whether the in vivo phenotypes observed in *Fgf21^−/−^* mice can be recapitulated ex vivo, we examined the effects of FGF21 using FTOC. Fetal thymi (E15.5) were cultured for 14 days in the presence of rFGF21. Consistent with findings in *Fgf21^−/−^* mice (Figs. 3 and 4), rFGF21 significantly increased clonal deletion and the number of Aire^+^ mTECs (fig. S7, A and B). In mTECs, rFGF21 increased the proportion of proliferating Ki67^+^ cells but did not affect the proportion of apoptotic cleaved caspase3^+^ cells (fig. S7, C and D). In the thymus, cDC1 differentiates from precursor cells, whereas cDC2 and pDC migrate to the thymus from peripheral tissues (*23*). In the FTOC, cDC1 accounted for approximately 2%, while the frequency of cDC2 and pDC was extremely limited due to the lack of migration from peripheral tissues (fig. S7E). rFGF21 did not alter cDC1 numbers but significantly upregulated MHC class I and II expression (fig. S7, E and F). These results parallel in vivo observations, indicating that secreted FGF21 acts within the thymus to sustain central immune tolerance.

### *Fgf21* deficiency reduces protein synthesis in mTECs

To explore the molecular basis of FGF21 function in mTECs, we performed RNA-sequencing (RNA-seq) on sorted mTECs from WT and *Fgf21^−/−^* mice. A total of 541 differentially expressed genes (DEGs) were identified (|fold change| ≥ 2, *P* < 0.05), with 257 up- and 284 downregulated in *Fgf21^−/−^* mTECs. In *Fgf21^−/−^* mTECs, the expression of *fucosyltransferase 1* (*Fut1*), an mTEC marker, was significantly reduced compared with its level in WT mTECs. The *Fgf21^−/−^* mice used in this study had most of the exon 1 and all exons 2 and 3 of the *Fgf21* gene replaced with an IRES-LacZ-polyA/PGK-neo cassette (*29*). *Fgf21* is located on mouse chromosome 7 and lies within several dozen kilobases of the neighboring gene *Fut1*. Because of this proximity, it is possible that enhancer or promoter regions near the targeted *Fgf21* locus were inadvertently affected, potentially leading to reduced *Fut1* expression in *Fgf21^−/−^* mTECs. Notably, the thymic phenotypes observed in *Fgf21^−/−^* mice were reproduced by blocking FGF21 with a neutralizing antibody in WT FTOC (fig. S7G) and were conversely reversed by rFGF21 supplementation (fig. S7, A to F), supporting the interpretation that these defects are attributable to impaired signaling by secreted FGF21 rather than to secondary consequences of genetic targeting at the Fgf21 locus.

**Fig. 6.**
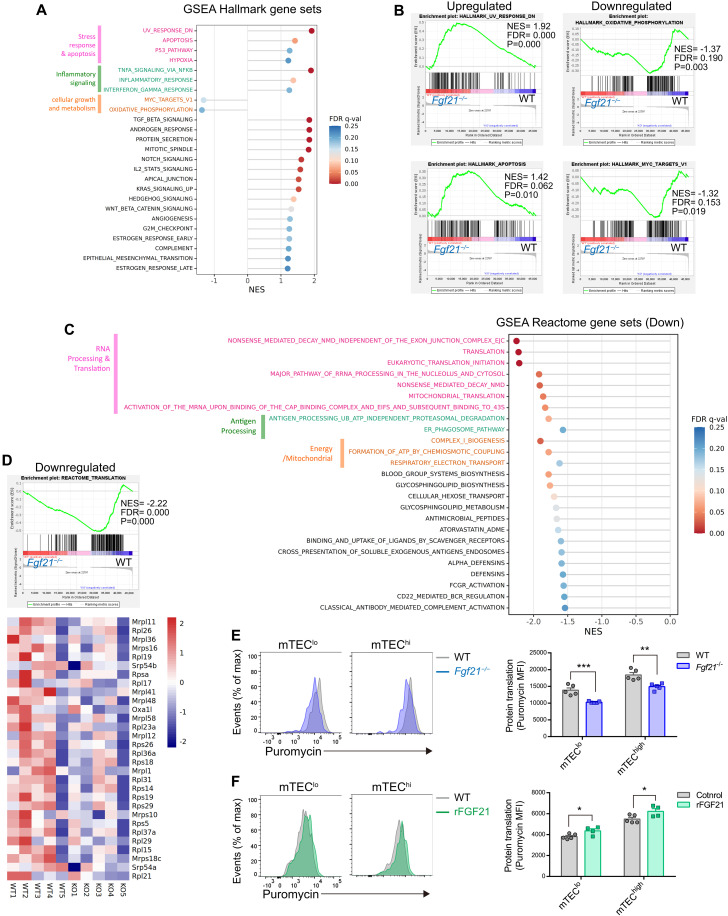
*Fgf21* deficiency attenuates protein synthesis in mTECs. (**A**) GSEA of HALLMARK pathways showing gene sets significantly enriched at FDR <0.25 in *Fgf21^−/−^* mTECs. (**B**) Representative enrichment plot from the HALLMARK analysis illustrating the normalized enrichment score for a selected pathway. (**C**) Reactome GSEA depicting downregulated gene sets (FDR < 0.25) in *Fgf21^−/−^* mTECs. (**D**) Reactome enrichment plot for the “TRANSLATION” pathway together with a heatmap of the 30 leading-edge genes contributing to the enrichment score. (**E** and **F**) Puromycin-incorporation assays in 1-month-old WT and *Fgf21^−/−^* mice (**E**) and in fetal thymic organ cultures (**F**) demonstrating reduced protein synthesis in *Fgf21^−/−^* mTECs (*n* = 4–5 per group). Two-tailed unpaired Student’s *t* tests s. *P < 0.05, **P < 0.01, ***P < 0.001.

GO analysis based on the DEG set did not identify any GO terms with significant enrichment. In contrast, gene set enrichment analysis (GSEA), which evaluates pathway-level changes across the entire ranked transcriptome rather than only the DEG subset, revealed distinct transcriptional alterations in *Fgf21^−/−^* mTECs. In the HALLMARK gene sets, pathways related to stress response and apoptosis (e.g., UV_RESPONSE_DN and APOPTOSIS) and inflammatory signaling were significantly upregulated, whereas cellular growth and metabolism pathways such as MYC_TARGETS_V1 and OXIDATIVE_PHOSPHORYLATION were downregulated (Fig. 6, A and B). In the Reactome gene sets, seven pathways associated with RNA processing and translation were markedly downregulated in *Fgf21^−/−^* mTECs (Fig. 6, C and D). In addition, pathways involved in antigen processing and mitochondrial energy metabolism were also suppressed in *Fgf21^−/−^* mTECs (Fig. 6C). These results indicate that FGF21 contributes to maintaining translational and metabolic homeostasis in mTECs, thereby supporting their capacity for protein synthesis and self-antigen presentation.

To evaluate the effect of FGF21 on translation in mTECs, we performed in vivo puromycin-incorporation experiments. As puromycin is an analog of ribosomal aminoacyl-tRNA, it is incorporated into nascent polypeptide chains (*30*). Puromycin uptake was significantly reduced in mTECs from *Fgf21^−/−^* mice but increased in FTOC supplemented with rFGF21 (Fig. 6, E and F). These findings suggest that FGF21 plays an important role in translation in mTECs.

### Endoplasmic reticulum stress-induced FGF21 suppresses the ATF4–CHOP apoptotic pathway

**Fig. 7.**
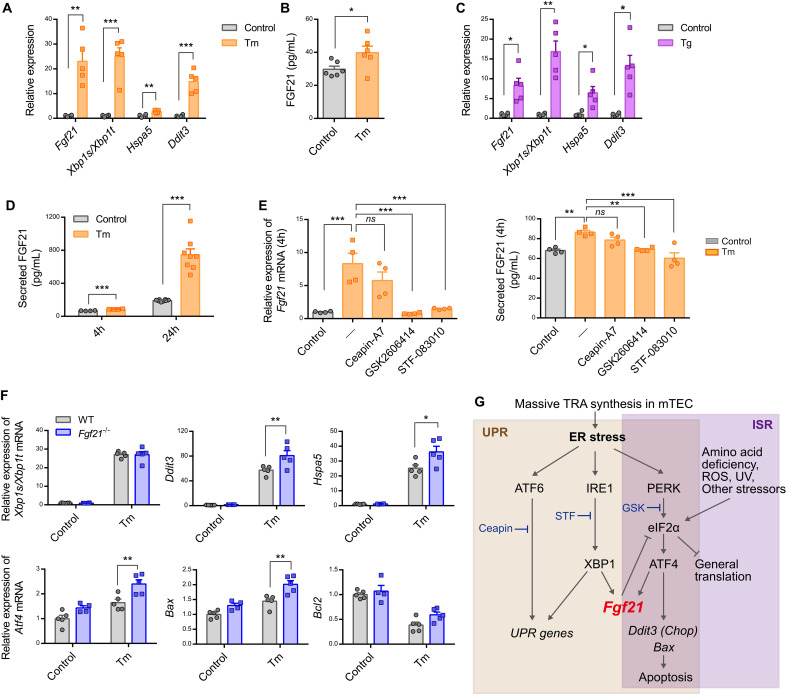
FGF21 is induced by ER stress and suppresses the ATF4-CHOP pathway. (**A** to **C**) 2DG-FTOC from E15.5 WT mice were stimulated with 5 μg/ml Tm or 200 nM Tg for 4 hours (*n* = 4–6). (**D**) TEC3–10 cells were treated with Tm and incubated for 4 (*n* = 4) or 24 hours (*n* = 8). (**E**) TEC3–10 cells were pretreated for 30 min with the inhibitors STF-083010 (60 μM), GSK2606414 (0.6 μM), or Ceapin-A7 (3 μM) and then stimulated with Tm for 4 hours (*n* = 4). (**F**) 2DG-FTOC from E15.5 WT or *Fgf21^−/−^* mice were stimulated with Tm for 24 hours (*n* = 4–5). (**G**) Schematic model summarizing ER stress–driven UPR/ISR signaling that induces FGF21 expression in mature mTECs to preserve proteostasis. For statistical analysis, two-tailed unpaired Student’s *t* tests [(A) to (D)], one-way ANOVA followed by Tukey’s post-hoc test (E), and two-way ANOVA followed by Tukey’s post-hoc test (F) were used. *P < 0.05, **P < 0.01, and ***P < 0.001; ns, not significant. h, hours.

As mTECs mature, massive TRA synthesis induces endoplasmic reticulum (ER) stress and activates the unfolded protein response (UPR) (*31*, *32*). In hepatocytes, FGF21 expression is regulated by pathways involved in the UPR, such as the IRE1α–XBP1 and PERK–eIF2α–ATF4 pathways (*33*, *34*). We hypothesized that increased ER stress in mature mTECs induces the expression of FGF21. When lymphocyte-depleted 2DG-treated FTOCs were treated with tunicamycin (Tm), which induces ER stress by inhibiting protein glycosylation, *Xbp1* splicing, and the expression of *Hspa5*, *Ddit3* (CHOP), and *Fgf21* were markedly increased, along with FGF21 protein secretion (Fig. 7, A and B). Thapsigargin (Tg), which induces ER stress by reducing chaperone function through ER Ca^2+^ depletion, also promoted the expression of UPR target genes and *Fgf21*, similar to Tm stimulation (Fig. 7C). Moreover, when ER stress was induced in the mTEC cell line (TEC3–10) by Tm stimulation, a significant increase was noted in both *Fgf21* mRNA and protein levels (Fig. 7D). Tm-induced FGF21 production was blocked by inhibitors of the IRE1α–XBP1 (STF-083010) and PERK–eIF2α–ATF4 (GSK2606414) pathways but not by the ATF6 inhibitor Ceapin-A7 (Fig. 7E), indicating that FGF21 is upregulated through these two UPR branches.

The integrated stress response (ISR) is a universal pathway that allows cells to adapt to various environmental stresses, including amino acid deprivation, oxidative stress, and ER stress (*35*). The ISR promotes cell adaptation via eIF2α phosphorylation and ATF4 activation, but prolonged activation induces CHOP, encoded by *Ddit3*, and apoptosis. In hepatocytes and cardiomyocytes, ER-stress-induced FGF21 suppresses the eIF2α-ATF4-CHOP pathway through negative feedback (*33*, *34*, *36*). When Tm was added to *Fgf21^−/−^* 2DG-FTOC, the expression of *Hspa5*, *Ddit3*, *Atf4*, and the pro-apoptotic molecule *Bax* significantly increased compared to that in WT, but there was no effect on the splicing of *Xbp1* mRNA or the expression of the anti-apoptotic molecule *Bcl2* (Fig. 7F). Together with increased apoptosis and reduced translation in *Fgf21^−/−^* mTECs (Figs. 4 and 6), these findings suggest that FGF21 protects mTECs by restraining the eIF2α–ATF4–CHOP pathway under ER stress (Fig. 7G).

To further define the cellular source and target of FGF21 within the thymic epithelial compartment, we sorted TECs into cTECs and three mTEC subsets based on MHC class II and CD80 expression: mTEC^lo^, CD80^int^ mTEC^hi^, and CD80^hi^ mTEC^hi^ subsets (fig. S8A) and examined the expression of *Fgf21* and its receptor components using RT-PCR. *Aire* mRNA expression increased stepwise from mTEC^lo^ to CD80^int^ mTEC^hi^ and CD80^hi^ mTEC^hi^ cells, confirming progressive maturation across these fractions (fig. S8B). *Fgf21* expression was low in mTEC^lo^ cells but was comparably elevated in both CD80^int^ mTEC^hi^ and CD80^hi^ mTEC^hi^ cells. *Fgfr1* was detected across mTEC subsets, with a slight decrease during maturation, whereas *Klb* expression was selectively detected in the CD80^hi^ mTEC^hi^ fraction. These findings suggested that *Fgf21* expression is induced during mTEC maturation, whereas responsiveness to FGF21 is greatest in the most mature *Klb*-expressing mTEC subset, in which FGF21 helps restrain excessive ISR activation and apoptosis.

## DISCUSSION

In this study, we demonstrated that FGF21 maintains central immune tolerance by preserving both the number and function of mTECs. mTECs uniquely express a broad repertoire of TRAs and play a dominant role in clonal deletion of self-reactive thymocytes (*37*). As mTECs mature, Aire-mediated TRA expression causes a high translational burden, which in turn triggers ER stress (*31*, *32*). FGF21 is induced in mTECs through the IRE1–XBP1 and PERK–eIF2α pathways under ER stress (Fig. 7).

ISR, triggered by the phosphorylation of eIF2α, is increasingly recognized as a central regulatory factor of protein homeostasis at both cellular and organismal levels (*38*). Activation of the eIF2α-ATF4 pathway in mature mTECs is thought to temporarily alleviate translational burden and prevent proteotoxicity. However, chronic ISR activation leads to sustained induction of the eIF2α–ATF4–CHOP signaling cascade, resulting in translational repression, diminished mitochondrial metabolism, impaired proliferation, and apoptosis (*32*, *38*, *39*). Consistent with this model, GSEA of *Fgf21^−/−^* mTECs revealed coordinated downregulation of pathways related to translation, mRNA processing, and mitochondrial energy metabolism (Fig. 6, A to D), indicating a global suppression of protein synthesis and metabolic activity that closely parallels sustained ISR activation (*35*). GSEA Reactome analysis revealed downregulation of the “ANTIGEN_PROCESSING_UB_ATP_INDEPENDENT_PROTEASOMAL_DEGRADATION” and “ER_PHAGOSOME_PATHWAY” gene sets (Fig. 6C), indicating partial impairment of proteasome- and ER-associated antigen processing. This may disrupt proteostasis, causing protein misfolding and ER stress in mTECs. Because proteasome function is essential for antigen presentation and mTEC maintenance (*31*, *40*), such an imbalance likely contributes to reduced mTEC number and function in *Fgf21^−/−^* mice. Furthermore, when ER stress was induced in *Fgf21^−/−^* 2DG-FTOC, the expression of *Atf4*, *Ddit3* (CHOP), and *Bax* was significantly increased compared to that in WT, consistent with previous reports that FGF21 suppresses the eIF2α-ATF4-CHOP pathway (*33*, *34*, *36*). Together, these findings indicate that FGF21 mitigates chronic ISR activation and thereby sustains protein synthesis, mitochondrial metabolism, and cell survival in mTECs.

Compared to the total number of developing T cells, the relatively small population of mTECs, combined with the mosaic and stage-restricted nature of TRA expression, significantly limits the T cell selection process (*3*, *41*). To address these constraints, TRAs from mTECs are transferred to DCs via CAT, allowing them to be indirectly presented to T cells (*23*, *24*, *42*). Although FGF21 did not directly act on DCs (figs. S5, A and B), *Fgf21* deficiency reduced MHC expression and pMHC transfer from mTECs to DCs (Fig. 5D), suggesting that impaired antigen relay limits clonal deletion. The decrease in mature mTECs (Fig. 4, A and B) and instability of MHC expression (Fig. 5E) in *Fgf21^−/−^* TECs likely underlie this defect. Additional mechanisms, such as altered chemokine signaling (e.g., XCL1–XCR1), may also contribute and warrant further study.

Conditional deletion of *Klb*, a co-receptor for FGF21, from Foxn1-origin TECs did not affect the total number of thymic cells in adult mice at 6 or 12 months of age but resulted in a significant decrease at 24 months of age (*15*). These results suggest that the physiological action of FGF21 on KLB-dependent TECs may be partially necessary but not essential for maintaining thymic function during aging. In 1-month-old *Fgf21^−/−^* mice, mTEC numbers were already reduced while total thymocytes increased owing to impaired clonal deletion (Figs. 3 to 4 and fig. S2). Physiological *Fgf21* deficiency reduced clonal deletion and the number of mature mTECs as early as 1 month of age, when thymic function is most developed (Figs. 3 and 4). In contrast, thymic involution and the accompanying decrease in thymic SP cell and splenic naïve T cell numbers were not observed (figs. S2 and S3). Given that autoimmune phenotypes became evident only in aged *Fgf21^−/−^* mice, we examined the thymus in 14-month-old mice. At this age, thymus weight and total thymocyte number remained comparable between WT and *Fgf21^−/−^* mice, indicating no gross difference in overall thymic size or cellularity (fig. S6A). Nevertheless, aged *Fgf21^−/−^* mice exhibited a reduced mTEC compartment, consistent with the defect observed in young mice (fig. S6, B and C). Together, these findings suggested that thymic defects caused by *Fgf21* deficiency persist with aging and may contribute to the later emergence of autoimmunity by impairing mTEC maintenance. In the thymus, *Klb* expression and rFGF21-induced ERK phosphorylation were observed only in mTECs, suggesting that FGF21 signaling preferentially acts within the mTEC compartment (fig. S5, A and B). Consistent with this observation, *Fgf21* expression increased during mTEC maturation, whereas *Klb* expression was most evident in the most mature mTEC^hi^ fraction (fig. S8), supporting a model in which FGF21 signaling is most relevant in mature mTECs. Thus, the physiological role of thymus-derived FGF21 appears to be maintenance of the number and function of mTECs from early life onward, thereby supporting clonal deletion and helping preserve central tolerance across the lifespan. Several studies have shown that age-related thymic involution is caused by changes in cTECs (*43*, *44*). It has been reported that overexpression of FGF21 in TECs acts in a paracrine manner to increase the number of cTECs and protect against thymic involution (*14*, *15*). Accordingly, our data support a model in which physiological FGF21 acts predominantly within the mTEC compartment, whereas higher levels of FGF21, such as in overexpression models, may additionally influence cTECs through paracrine or KLB-independent mechanisms. Further clarification of FGF21 regulation of cTECs will be important for understanding its broader protective role in thymic aging.

Thymus-transplantation experiments demonstrated that thymus-derived FGF21 prevents peripheral autoimmunity (Fig. 1, C and D). Limitations include the use of systemic *Fgf21^−/−^* mice, which prevents cell-type-specific evaluation, and the lack of temporal analysis of FGF21 function. Future studies using conditional or inducible knockouts will help determine the developmental window and target cells of FGF21 action.

**Fig. 8.**
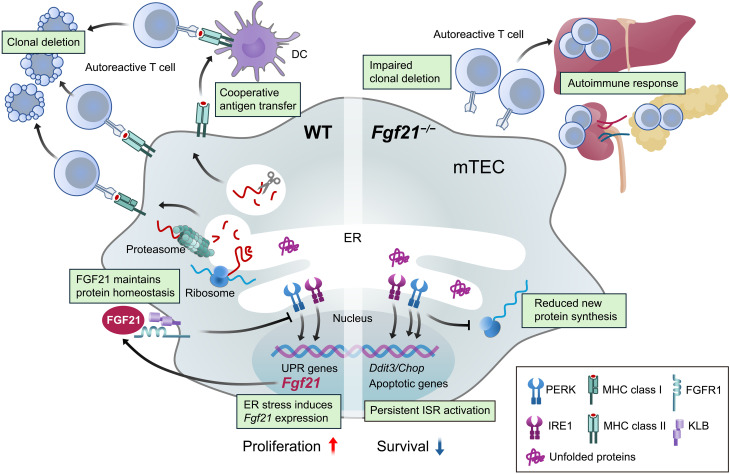
FGF21 preserves central immune tolerance by maintaining mTEC proteostasis and function. Schematic model illustrating the role of FGF21 in mTECs. In WT mice, ER stress during mTEC maturation activates the IRE1–XBP1 and PERK–eIF2α pathways, leading to the induction of *Fgf21* expression. Together with the predominant expression of the co-receptor *Klb* in the most mature mTEC^hi^ population, this suggests that FGF21 signaling preferentially acts within mature mTECs. FGF21 maintains protein homeostasis by limiting sustained activation of the PERK–eIF2α–ATF4 pathway, thereby supporting protein synthesis, proliferation, and survival. This preserves antigen processing and presentation, cooperative antigen transfer to DCs, and efficient clonal deletion of autoreactive T cells. In *Fgf21^−/−^* mice, sustained ER stress results in persistent activation of the PERK–eIF2α–ATF4 branch of the ISR, leading to reduced new protein synthesis and induction of *Ddit3/Chop* and apoptotic genes. Consequently, mTEC number and function are reduced, antigen transfer to DCs is impaired, and autoreactive T cells escape negative selection, leading to autoimmune responses in peripheral tissues.

In summary, FGF21 is induced by ER stress during mTEC maturation and functions to limit sustained ISR, thereby maintaining protein synthesis, proliferation, and survival of mTECs. By preserving mTEC integrity and promoting cooperation with thymic DCs, FGF21 supports efficient clonal deletion and central immune tolerance (Fig. 8). Together, these findings support a model in which the thymic FGF21 axis safeguards central tolerance and protects against autoimmunity and highlight this pathway as a potential therapeutic target for immune-mediated disorders.

## MATERIALS AND METHODS

### Experimental Design

This study aimed to determine whether FGF21 produced by mTECs contributes to central immune tolerance. Autoimmune manifestations and clonal deletion were assessed in WT and *Fgf21^−/−^* mice. To evaluate the physiological role of thymic FGF21, fetal thymi from WT or *Fgf21^−/−^* embryos were transplanted into athymic nude mice, and autoimmune responses were analyzed. The effects of FGF21 on mTECs and thymic DCs were examined through phenotypic and functional analyses using thymus and FTOC from WT and *Fgf21^−/−^* mice. Mechanisms of FGF21 induction were investigated in lymphocyte-depleted 2DG-FTOCs and the mTEC line TEC3–10 under Tm stimulation.

### Mice

WT C57BL/6 mice and C57BL/6 background *Fgf21^−/−^* mice were generated as described previously (*29*). Female mice aged 14 months were used for autoimmune studies, and 1-month-old females were used for other experiments. Female athymic BALB/c nu/nu were purchased from CLEA Japan (Tokyo, Japan). All animal procedures were conducted in accordance with the Guidelines for the Care and Use of Laboratory Animals of Kobe Pharmaceutical University (Kobe, Japan) and were approved by the institutional ethics committee (protocol nos. 2020–057, 2021–056, 2022–033, 2023–051, and 2024–042). Mice were euthanized by isoflurane anesthesia followed by cervical dislocation, and every effort was made to minimize suffering.

### Flow cytometry

Single-cell suspensions of thymus and spleen were prepared as described in the Supplementary Materials. Surface and intracellular staining were performed using standard protocols with fluorochrome-conjugated antibodies (see Supplementary Materials, *Flow cytometry*). Data were acquired on a FACSAria III (BD Biosciences, San Jose, CA, USA) and analyzed with FlowJo software (FlowJo, LLC, Ashland, OR, USA).

### BrdU incorporation assay

Four-week-old WT or *Fgf21^−/−^* mice received an intraperitoneal injection of 1 mg BrdU (eBioscience, San Diego, CA, USA). After 24 hours, thymic single-cell suspensions were prepared and stained for surface antigens. BrdU incorporation was detected using a BrdU Staining Kit (eBioscience) following the manufacturer’s instructions.

### FTOC and thymic transplantation

Thymic lobes were isolated from E15.5 embryos and cultured on Millicell 0.4-μm inserts (Merck Millipore, Darmstadt, Germany) in serum-free AIM-V medium (Thermo Fisher Scientific, Waltham, MA, USA) with or without 500 ng/ml recombinant human FGF21 or 2 μg/ml anti-mouse FGF21 antibody (R&D Systems, Minneapolis, MN, USA). The medium was replaced every 3 days, and after 14 days, cells were analyzed by flow cytometry.

For 2-deoxyguanosine (2DG)–treated FTOC, thymic lobes from E15.5 WT or *Fgf21^−/−^* embryos were cultured for 5 days with 1.35 mM 2DG (Sigma-Aldrich, St. Louis, MO, USA). After washing with phosphate-buffered saline (PBS), lobes were transplanted under the kidney capsule of 8-week-old female BALB/c nude mice. T cell reconstitution was confirmed by flow cytometry 10 weeks post-transplantation, and recipient organs were collected for histological analysis.

### Histological analysis

Lungs, stomach, liver, pancreas, and kidneys collected from 14-month-old WT or *Fgf21^−/−^* mice and from nude mice transplanted with fetal 2DG-treated thymus from WT or *Fgf21^−/−^* mice were fixed in 4% paraformaldehyde, embedded in paraffin blocks, and sectioned at 8 μm thickness. Sections were stained with hematoxylin and eosin (H&E) and examined by light microscopy.

### EAE

EAE was induced in 8-week-old WT and *Fgf21^−/−^* mice by immunization with MOG_35–55_ peptide as described previously. Disease severity was monitored daily using standard clinical scoring. Detailed procedures for immunization, clinical scoring, and tissue processing are provided in the Supplementary Materials.

### Intercellular MHC-transfer assay

An in vitro co-culture system using MHC-mismatched cells was performed as described previously (*26*). Stromal cells (CD45^−^) were isolated from WT and *Fgf21^−/−^* mice on a C57BL/6 background (H2^b^) by negative immunoselection using anti-mouse CD45 microbeads (Miltenyi Biotec; Bergisch Gladbach, Germany). Dendritic cells (DCs; CD11c^+^) were isolated from BALB/c (H2^d^) thymus by positive immunoselection using anti-mouse CD11c microbeads (Miltenyi Biotec). Stromal cells (1 × 10^6^ cells/ml) and DCs were mixed at a 1:1 ratio in U-bottom 96-well plates, co-cultured for 4 hours, and analyzed by flow cytometry.

### Enzyme-linked immunosorbent assays

FGF21 levels in serum or cell culture supernatants were analyzed using the enzyme-linked immunosorbent assay (ELISA) according to the manufacturer’s instructions (R&D Systems). The levels of antinuclear antibodies in the serum were analyzed by ELISA according to the manufacturer’s instructions (MyBioSource, San Diego, CA, USA).

### Reverse transcription-quantitative polymerase chain reaction (RT-qPCR)

Total RNA was extracted from tissues or cells using Sepasol-RNA I Super G (Nacalai Tesque, Kyoto, Japan). cDNA was synthesized with the ReverTra Ace qPCR RT kit, and qPCR was performed with Thunderbird SYBR qPCR mix (both from TOYOBO, Osaka, Japan). 18S rRNA served as the internal control. Primer sequences are listed in table S1 (Thermo Fisher Scientific).

### RNA-sequencing analysis

mTECs (CD45^−^EpCAM^+^Ly-51^−^) were isolated from WT and *Fgf21^−/−^* thymi by fluorescence-activated cell sorting. Total RNA was extracted and sequenced as 100-bp paired-end reads on a NovaSeq 6000 platform (Illumina, San Diego, CA, USA). Quality control, alignment, transcript assembly, and differential expression analyses were performed using standard RNA-seq pipelines. Detailed methods for sequencing, data processing, and enrichment analysis are provided in the Supplementary Materials.

### Cells

TEC3–10 (Riken Cell Bank, Tsukuba, Japan) cells (*45*–*47*) were cultured in Minimum Essential Medium (Nacalai Tesque) supplemented with 10% fetal bovine serum (Sigma-Aldrich), 5 μg/ml penicillin, 10 μg/ml streptomycin, and 0.5 ng/ml-hydrocortisone hemisuccinate (Nacalai Tesque).

### Measurement of protein synthesis

For in vivo analysis, puromycin (50 mg/kg) was administered intraperitoneally to 4-week-old mice, and 1 hour later, the mice were euthanized. In the FTOC, puromycin (10 μg/ml) was added and incubated at 37°C for 30 min. Single-cell suspensions were prepared from the thymus and stained with antibodies against cell-surface markers. Afterwards, the cells were fixed, permeabilized, and stained with PE-anti-puromycin antibody (BioLegend, San Diego, CA, USA).

### Statistical analysis

Data were analyzed using Prism software (GraphPad Software, San Diego, CA, USA) and are presented as mean + SD. Statistical tests used for each experiment are described in the corresponding figure legends. In general, two-group comparisons were performed using unpaired two-tailed Student’s *t*-tests, and multiple-group comparisons were analyzed by ANOVA followed by Tukey’s post-hoc test. *P* value < 0.05 was considered statistically significant.

## Supplementary Materials

### The PDF file includes:

Supplementary Text Figs. S1 to S8 Table S1 Legend for data S1 References
