# Supplementary material for: FGF21 induced by endoplasmic reticulum stress maintains medullary thymic epithelial cell function and central immune tolerance
Source: Sci Adv. 2026 Jul 17;12(29):eaee9999. doi: 10.1126/sciadv.aee9999 (PMC13378567; doi:10.1126/sciadv.aee9999)
Supplement: Supplementary file 1 — Supplementary Text Figs. S1 to S8 Table S1 Legend for data S1 References [file sciadv.aee9999_sm.pdf]

Science Advances
AAAS

Supplementary Materials for

FGF21 induced by endoplasmic reticulum stress maintains medullary thymic epithelial cell function and central immune tolerance

Yuki Masuda *et al.*

Corresponding author: Morichika Konishi, mkonishi@kobepharma-u.ac.jp

Sci. Adv. **12**, eaee9999 (2026)
DOI: 10.1126/sciadv.aee9999

The PDF file includes:

Supplementary Text
Figs. S1 to S8
Table S1
Legend for data S1
References

Other Supplementary Material for this manuscript includes the following:

Data S1

Supplementary Text

Materials and Methods

Flow cytometry (detailed methods)

For T-cell analysis, thymocytes and splenocytes were prepared by applying pressure to the thymus and spleen using the tip of a syringe and collected through a 70 μm cell strainer. For thymic epithelial cell (TEC) analysis, the thymus was digested with DNase I (Sigma-Aldrich, St. Louis, MO, USA) and Liberase^{TM} (Roche, Basel, Switzerland) to prepare a single-cell suspension. Staining of cell surface antigens with fluorescently labeled antibodies was performed in staining medium at 4°C for 20 min. Each isotype was then incubated under identical conditions. TCR Vβ staining was performed using the Anti-Mouse TCR Vβ Screening Panel (BD Pharmingen, San Diego, CA, USA).

To detect intracellular cytokines, the cell suspension was stimulated at 37°C for 4 h in RPMI 1640 medium containing 50 ng/mL phorbol 12-myristate 13-acetate (Sigma-Aldrich), 1 μg/mL ionomycin (Enzo Life Sciences, Farmingdale, NY, USA), and GolgiStop (1:1500; BD Pharmingen, San Diego, CA, USA). After stimulation, intracellular cytokines were stained using a BD Cytofix/Cytoperm Fixation/Permeabilization Kit (BD PharMingen). Nuclear protein staining was performed using the Foxp3 Transcription Factor Staining Buffer Set (eBioscience, San Diego, CA, USA).

The following antibodies were used: anti-CD45 (clone 30-F11), anti-CD25 (clone PC61), anti-CD5 (clone 53-7.3), anti-EpCAM (clone G8.8), anti-Ly51 (clone 6C3), anti-CD11c (clone N418), anti-PDCA-1 (clone 129c1), anti-Sirpα (clone P84), anti-MHC II (I-A/I-E) (clone M5/114.15.2), anti-CD80 (clone 16-10A1), anti-CD86 (clone GL1), anti-I-Ad (clone 39-10-8), anti-I-Ab (clone AF6-120.1), anti-CD36 (clone HM36), anti-IL-17A (clone TC11-18H10), anti-Ki67 (clone 16A8), anti-Puromycin (clone 2A4), and anti-CD73 (clone TY/11.8; all from BioLegend, San Diego, CA, USA); anti-CD8α (clone 53-6.7), anti-CD4 (clone RM4-5), anti-TCRβ (clone H57-597), anti-CCR7 (clone 4B12), anti-CD69 (clone H1.2F3), anti-CD62L (clone MEL-14), anti-CD44 (clone IM7), anti-ERK1/2 (pT202/pY204; clone 20A), anti-interferon (IFN)-γ (XMG1.2), anti-cleaved caspase 3 (C92-605.rMAb), anti-Foxp3 (clone MF23; BD PharMingen, San Diego, CA, USA), and anti-Aire (clone 5H12; Thermo Fisher Scientific, Waltham, MA, USA).

Phosphorylation flow cytometry

A single-cell suspension from the thymus of 1-month-old wild-type (WT) mice was incubated in RPMI with 10% FBS at 37°C for 2 h and then stimulated with rFGF21 (200 ng/mL) for 15 min. After stimulation, cells were fixed with Lyse/Fix buffer (BD Biosciences, San Diego, CA, USA) and permeabilized with cold BD Phosflow Perm Buffer II (BD Biosciences). The cells were then stained with surface antigen antibodies and Phospho-p44/42 MAPK (Erk1/2) Thr202/Tyr204 antibodies (BioLegend). Subsequently, analysis was conducted using a flow cytometer.

RNA sequencing analysis (detailed methods)

Medullary thymic epithelial cells (mTECs; $CD45^{-}EpCAM^{+}Ly\text{-}51^{-}$) were isolated from thymic single-cell suspensions of WT or *Fgf21*$^{-/-}$ mice by fluorescence-activated cell sorting (FACSAria III, BD Biosciences). Sorted mTECs were preserved in RNAlater (Thermo Fisher Scientific), and total RNA was extracted using the RNeasy Mini Kit according to the manufacturer's protocol (QIAGEN, Hilden, Germany). RNA quality and integrity were verified using an Agilent

Bioanalyzer. RNA-seq libraries were prepared and sequenced on an Illumina NovaSeq 6000 platform (Illumina, San Diego, CA, USA) to generate 100-bp paired-end reads. Raw sequencing reads were assessed with FastQC, and adapter sequences, low-quality bases, and contaminant reads were trimmed using Trimmomatic (v0.39) (*48*). Clean reads were aligned to the mouse reference genome (GRCm39) using the splice-aware aligner HISAT2 (v2.2.1) (*49*), and transcript assembly and quantification were performed with StringTie (v2.2.1) (*50*). Read counts were imported into R and analyzed with DESeq2 (v1.38.0) (*51*) to identify differentially expressed genes between WT and *Fgf21*$^{-/-}$ mTECs. Genes with |fold change| ≥2 and $P<0.05$ were considered significant. Gene Set Enrichment Analysis (GSEA) was conducted with HALLMARK and Reactome gene sets obtained from the Molecular Signatures Database (MSigDB v2023.1)(*52*). GSEA was applied to a preranked list of all expressed genes without applying arbitrary cutoffs, allowing detection of statistically significant enrichment in functionally related gene sets.

Experimental autoimmune encephalomyelitis (detailed methods)

EAE was induced by myelin oligodendrocyte glycoprotein (MOG) peptide immunization, as previously described (*53*). An emulsion was formulated by thoroughly combining 200 μg of MOG35-55 peptide (Biorbyt Ltd., Cambridge, UK) with Complete Freund's Adjuvant containing inactivated Mycobacterium tuberculosis H37Ra (Difco Laboratories, Detroit, MI, USA). Subsequently, the emulsified peptide was administered subcutaneously (200 μL) to the dorsal region of 8-week-old WT and *Fgf21*$^{-/-}$ mice. On the day of immunization and one day later, 200 ng of pertussis toxin (List Biological Laboratories, Campbell, CA, USA) was administered intraperitoneally. Mice were monitored for the development of clinical symptoms over time. 0, normal; 1, reduced tail tone; 2, complete tail paralysis; 3, abnormal gait; 4, complete paralysis of the hind limbs; 5, complete paralysis of the hind limbs, including forelimb paralysis; 5, moribund state. The liver and thymus were collected on days 4, 8, 14, and 18, and mRNA expression of *Fgf21* was measured. Twenty-one days post-immunization, the spinal cords were extracted from the mice, minced, and treated with collagenase. The tissue was then passed through a cell strainer and centrifuged at 300g for 5 min. Ten milliliters of 38% Percoll solution was added to the pellet and gently mixed, followed by centrifugation at 800×g for 30 min at room temperature (with brake off). The cell pellet was subsequently subjected to flow cytometry.

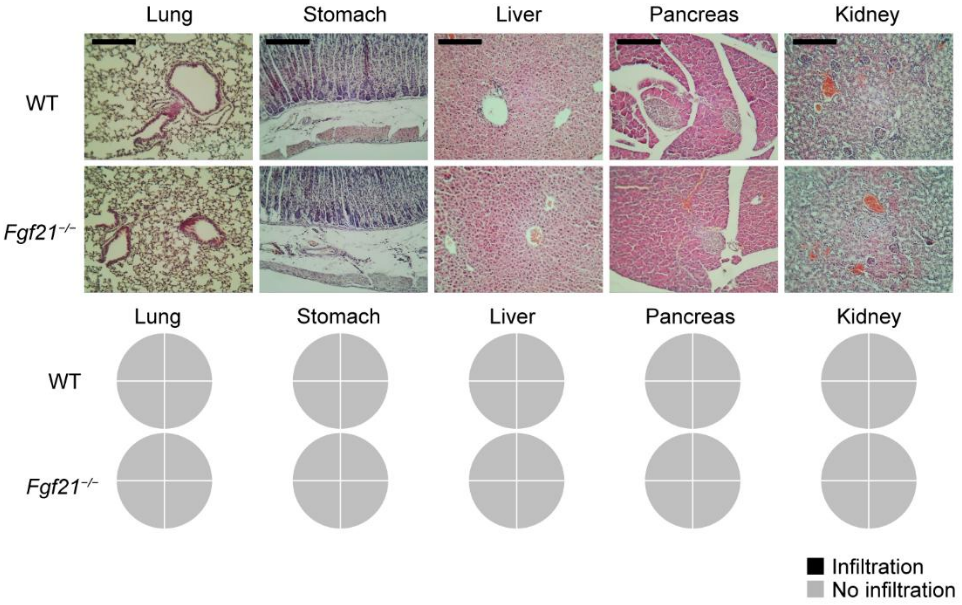

Fig. S1. Young *Fgf21*-deficient mice do not show autoimmune responses.

Representative hematoxylin and eosin (H&E)-stained images of the lungs, stomach, liver, pancreas, and kidneys from 2-month-old WT (n = 4) and *Fgf21*$^{-/-}$ (n = 4) mice. No histological evidence of autoimmune responses was observed in any examined tissues from either group. Scale bar, 200 μm.

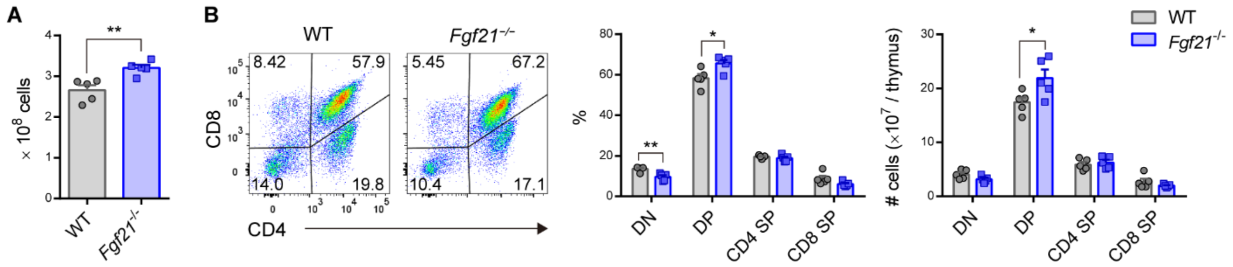

Fig. S2. Effect of *Fgf21* deficiency on the thymic T cell population.

(A) Total cell numbers in the thymus of 1-month-old WT and *Fgf21*$^{-/-}$ mice (n=12–13). **(B)** Frequency and number of CD4^{-}CD8^{-} (DN), CD4^{+}CD8^{+} (DP), CD4^{+}CD8^{-}(CD4 SP), and CD4^{-}CD8^{+} (CD8 SP) cells (n=12–13). Two-tailed unpaired Student's t-tests were used for statistical analysis.

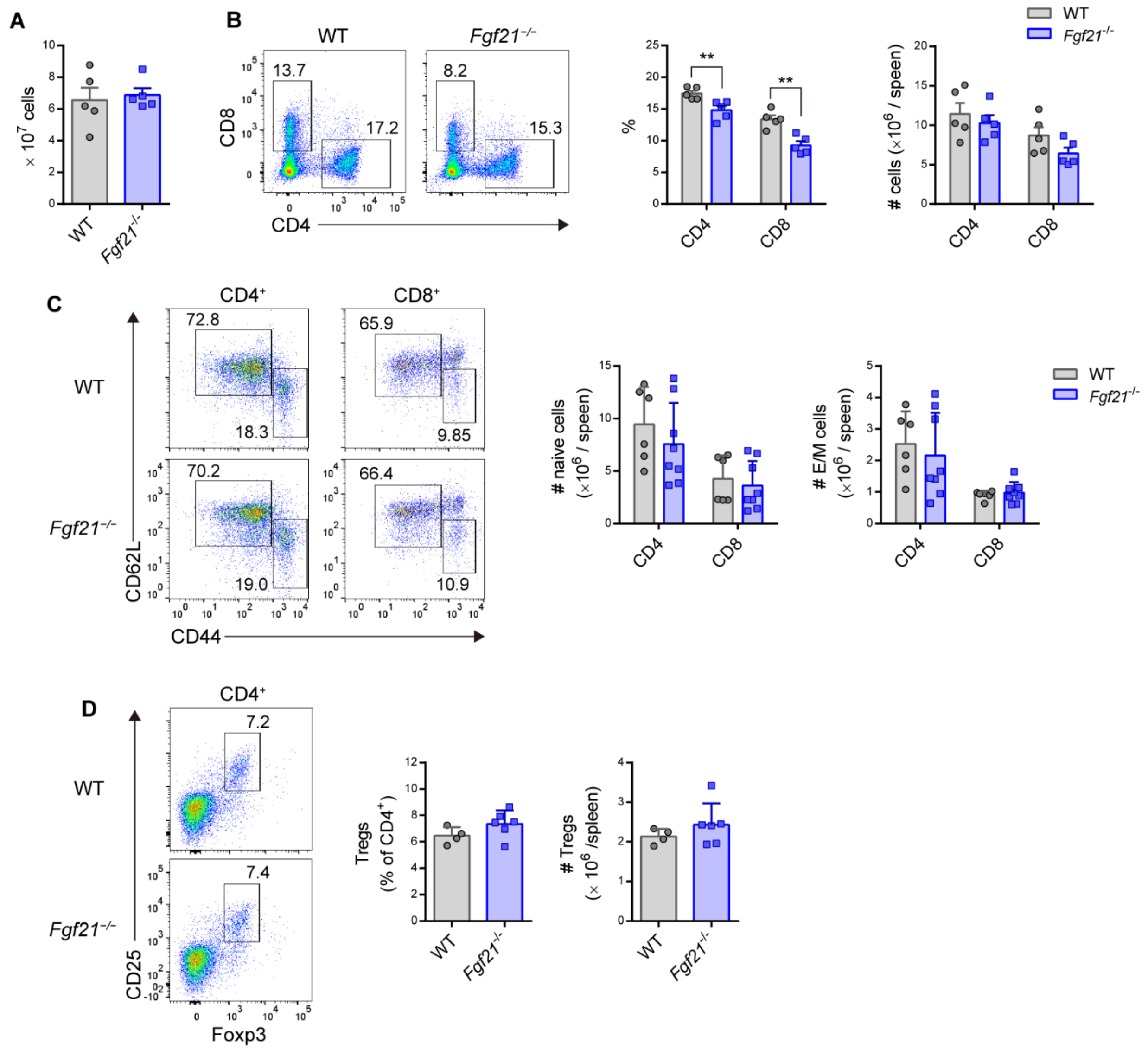

Fig. S3. Effect of *Fgf21* deficiency on splenic T cell populations.

(A) Total cell number in the spleens of 1-month-old WT and *Fgf21*$^{-/-}$ mice (n=12–13). **(B)** Frequency and number of $CD4^{+}$ and $CD8^{+}$ T cells (n=12–13). **(C)** Number of naïve ($CD62L^{+}CD44^{-}$) and effector/memory (E/M; $CD62L^{-}CD44^{+}$) cells (n=6–7). **(D)** Frequency of Tregs in $CD4^{+}$ T cells and number of Treg cells in the spleen (n=4–6). Two-tailed unpaired Student's t-tests were used for statistical analysis. **P<0.01.

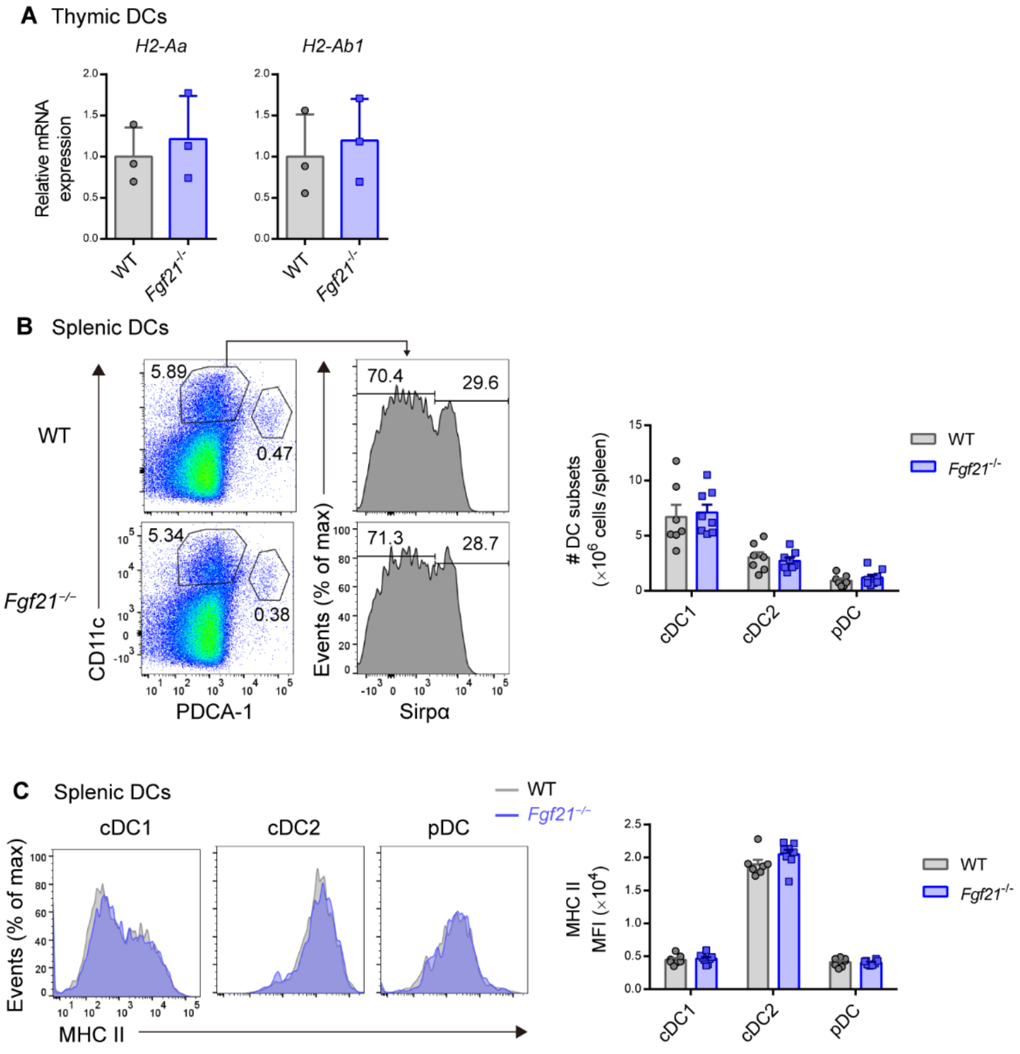

Fig. S4. Effects of *Fgf21* deficiency on splenic and thymic DCs.

(A) DCs were isolated from the thymus of one-month-old WT and *Fgf21*$^{-/-}$ mice using CD11c microbeads, and the relative mRNA levels of *H2-Aa* and *H2-Ab1* were determined by RT-qPCR (n=3). **(B)** Flow cytometry profile of splenic DCs. Cell numbers of cDC1 (CD11c^{+}PDCA^{-} Sirpα^{-}), cDC2 (CD11c^{+}PDCA^{-}Sirpα^{+}), and pDC (CD11c^{-}PDCA^{+}) (n=7–8). **(C)** Expression levels of MHC class II molecules in each splenic DC subset. Representative flow cytometry data and mean fluorescence intensity (MFI) are shown (n=7–8). Representative flow cytometry data and MFI are shown (n=6). For statistical analysis, two-tailed unpaired Student's t-tests were used.

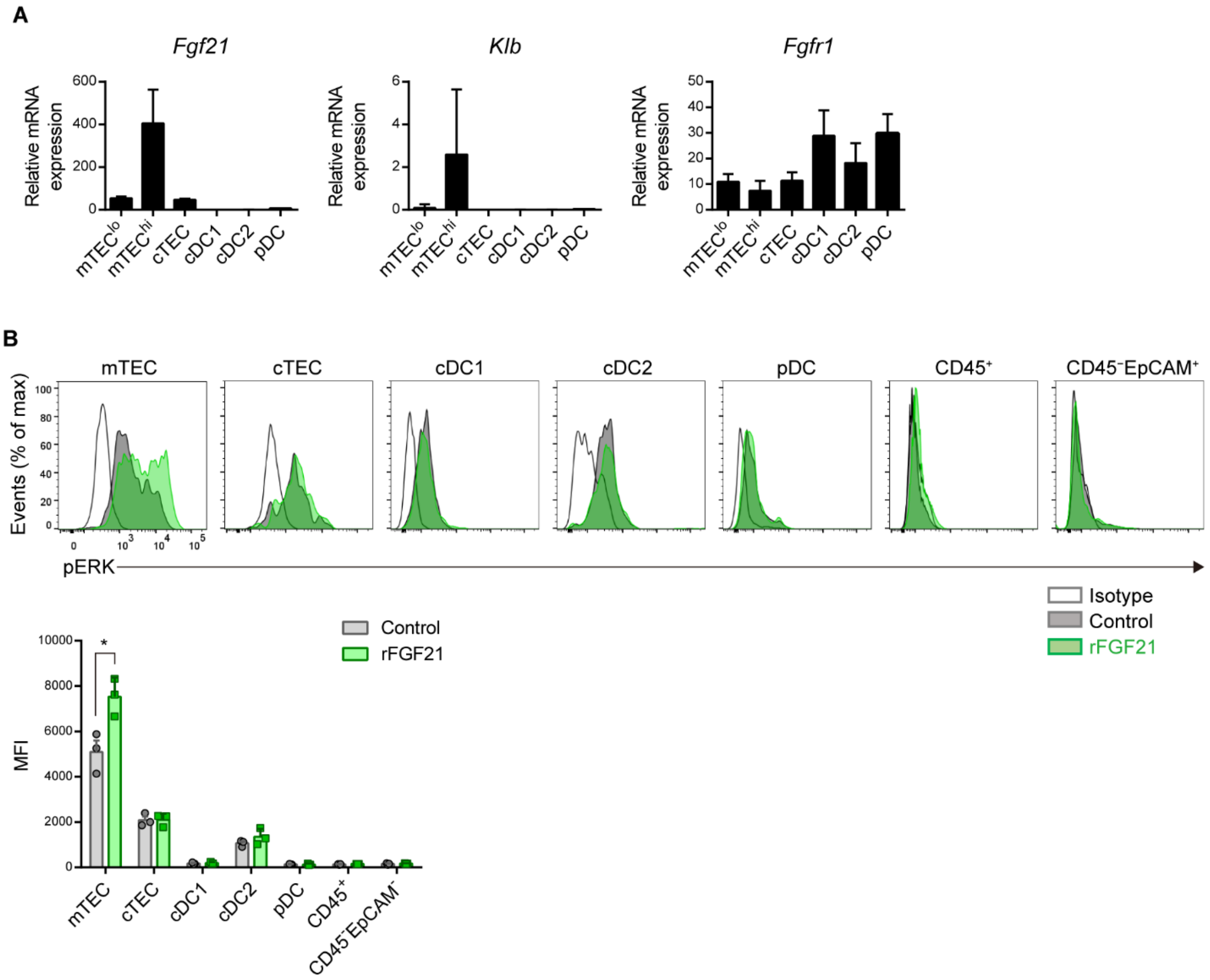

Fig. S5. Expression of FGF21 receptors and rFGF21-induced ERK phosphorylation.

(A) The expression levels of *Fgf21*, *Fgfr1*, and *Klb* were compared in mTEC^{lo} (CD45$^{–}$EpCAM^{+}Ly-51$^{–}$MHC II^{lo}), mTEChi (CD45$^{–}$EpCAM^{+}Ly-51$^{–}$MHC II^{hi}), cTEC (CD45$^{–}$EpCAM^{+}Ly-51^{+}), cDC1 (CD11c^{+}PDCA$^{–}$Sirpα$^{–}$), cDC2 (CD11c^{+}PDCA$^{–}$Sirpα^{+}), and pDC (CD11c$^{–}$PDCA^{+}) (n=4–5) sorted from thymic single-cell suspensions of 1-month-old WT mice. **(B)** rFGF21 (200 ng/mL) was added to thymic single-cell suspensions from 1-month-old WT mice and incubated at 37°C for 15 min. After fixation and permeabilization, cells were stained with a fluorescent antibody targeting pERK (n=3). Two-tailed unpaired Student's t-tests were used for statistical analysis. *P<0.05.

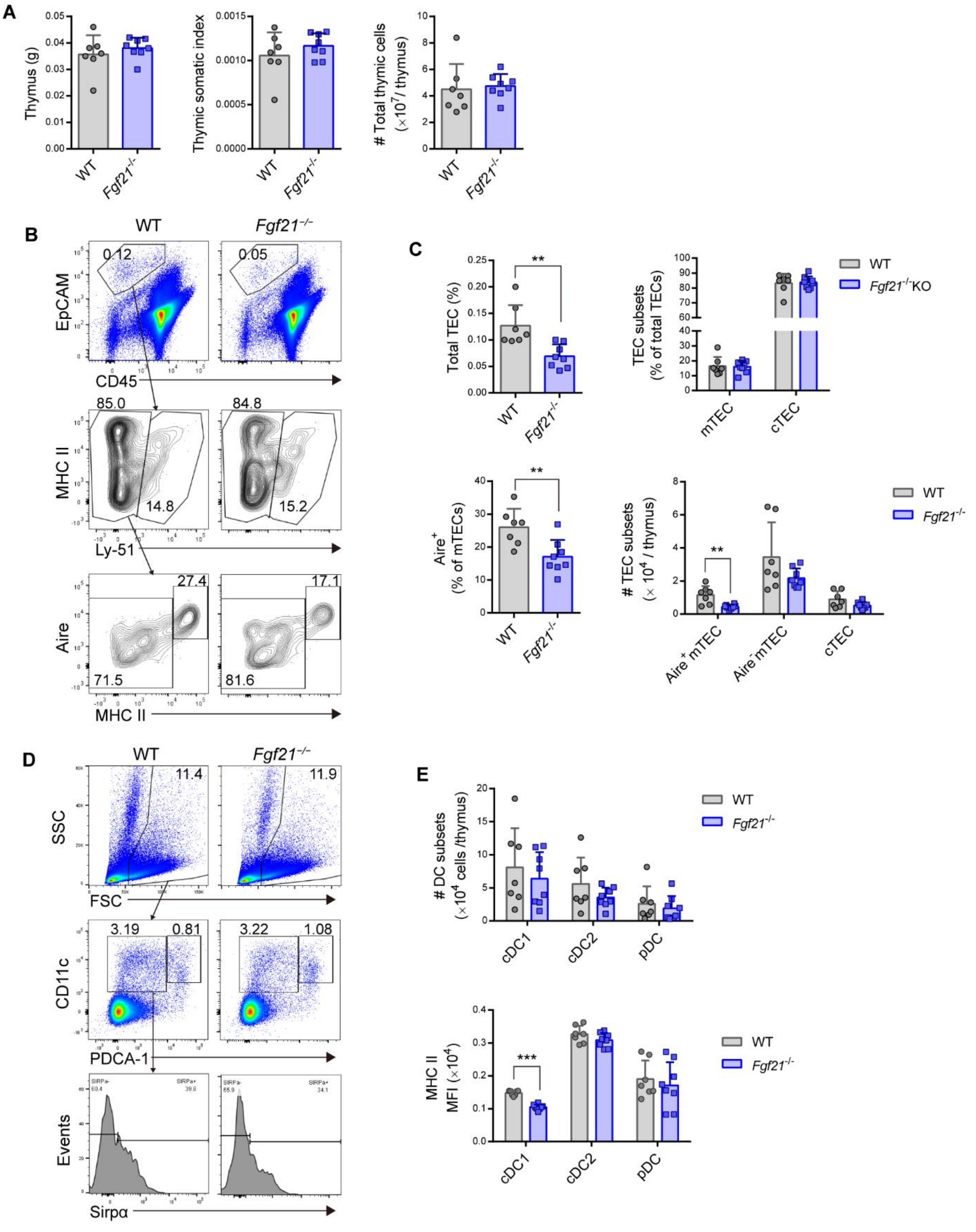

Fig. S6. Thymic epithelial and dendritic cell analysis in aged *Fgf21*$^{–/–}$ mice.

(A–E) Thymi from 14-month-old WT (n=7) and *Fgf21*$^{–/–}$ (n=8) mice were analyzed. **(A)** Thymus weight, thymic somatic index (thymus weight normalized to body weight), and total thymocyte numbers. **(B)** Flow cytometry gating strategy for TECs. **(C)** Frequencies of TECs, mTECs and

cTECs among TECs, and Aire$^+$ cells among mTECs, are shown. Cell numbers of Aire$^+$ mTECs, Aire$^-$ mTECs, and cTECs per thymus are shown. **(D)** Flow cytometry gating strategy for thymic DCs. **(E)** Cell numbers of thymic DC subsets (cDC1, cDC2, and pDC) per thymus and their MHC class II expression levels (MFI). Two-tailed unpaired Student's t-tests were used for statistical analysis. $^{**}P < 0.01$, $^{***}P < 0.001$.

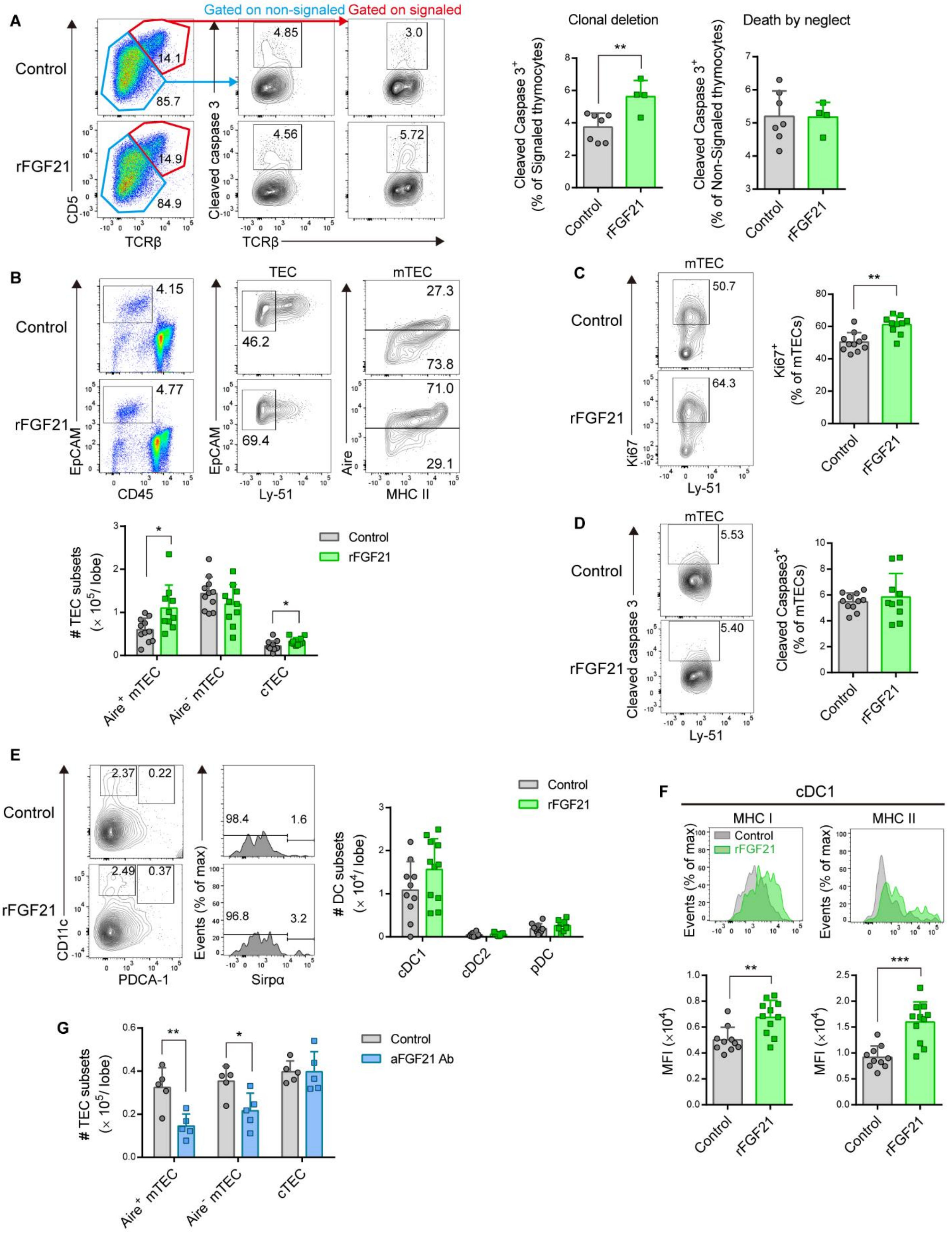

A
Gated on non-signaled
Gated on signaled
Control
rFGF21
CD5
TCRβ
Cleaved caspase 3
TCRβ
Clonal deletion
Death by neglect
Cleaved Caspase 3+ (% of Signaled thymocytes)
Cleaved Caspase 3+ (% of Non-Signaled thymocytes)
B
TEC
mTEC
EpCAM
CD45
Ly-51
Aire
MHC II
TEC subsets (× 10^5/ lobe)
Aire+ mTEC
Aire- mTEC
cTEC
C
Ki67
Ki67+ (% of mTECs)
D
Cleaved Caspase3+ (% of mTECs)
E
CD11c
PDCA-1
Events (% of max)
Sirpα
DC subsets (× 10^4/ lobe)
cDC1
cDC2
pDC
F
MHC I
MHC II
MFI (×10^4)
G
aFGF21 Ab

Fig. S7. In FTOC, rFGF21 increased the number of mature mTECs and promoted clonal deletion.

(A-F) Thymi from E15.5 WT mice were cultured for 14 days in the presence or absence of rFGF21 (500 ng/mL) (n=10–11). **(A)** Flow cytometry gating strategy to distinguish death by neglect and clonal deletion. Signaled and non-signaled thymocytes were identified by the expression of CD5 and TCRβ. Death by neglect and clonal deletion was identified by the expression of cleaved caspase 3. **(B)** Flow cytometry profiles of the TECs. Frequency and number of $Aire^{+}$ mTECs, $Aire^{-}$ mTECs, and cTECs. Representative flow cytometry profiles and quantitative data of $Ki67^{+}$ mTECs and cTECs **(C)** and cleaved caspase 3^{+} mTECs and cTECs **(D)** are shown. **(E)** Flow cytometry profile of thymic DCs. Cell numbers of cDC1, cDC2, and pDC. **(F)** Expression levels of MHC class I and II in the thymic cDC1 subset. Representative flow cytometry data and MFI are shown. **(G)** Thymi from E15.5 WT mice were cultured for 14 days in the presence or absence of an anti-FGF21 polyclonal antibody (2 μg/mL) (n=5). The cell numbers of $Aire^{+}$ mTECs, $Aire^{-}$ mTECs, and cTECs are shown. Two-tailed unpaired Student's t-tests were used for statistical analysis. *P<0.05, **P<0.01, and ***P<0.001.

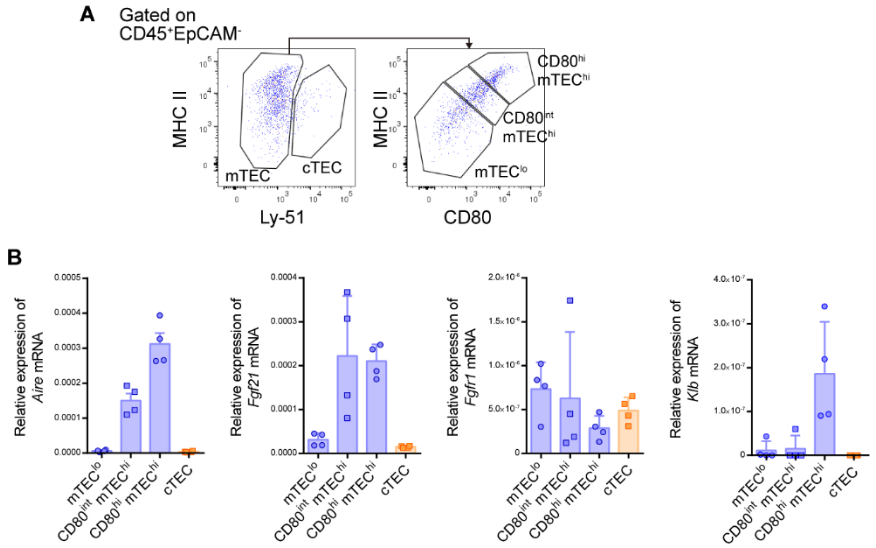

Fig. S8. Expression of *Fgf21* and its receptor components in mTEC subsets.

(A) Flow cytometric sorting of thymic epithelial cells into cTECs, mTEC^{lo}, CD80^{int} mTEC^{hi}, and CD80^{hi} mTEC^{hi} populations. **(B)** RT-PCR analysis of *Aire*, *Fgf21*, *Fgfr1*, and *Klb* expression in each subset (n=4).

Table S1. Primer sequences for RT-qPCR.

Gene name	Forward primer (5′–3′)	Reverse primer (5′–3′)
18s	CCGGAATCGAACCCTGATT	CGAACGTCTGCCCTATCAACTT
H2-Aa	CTGAACACCATGCTCAGCCTCT	TACTGGCCAATGTCTCCAGGAG
H2-Ab1	ACCCAGCCAAGATCAAAGTGC	TGCTCCACGTGACAGGTGTAGA
Fgf21	CCTGGGTGTCAAAGCCTCTA	CTCCAGCAGCAGTTCTCTGA
Fgfr1	TTGGAGGCTACAAGGTTCGC	GCGGATCGCTGTACACCTTA
Klb	ACGGCTGGTTCACAGATAGCT	CATCAAATTTTATTGCTTGAAGAACCT
Xbp1	TGACGAGGTTCCAGAGGTG	TGTTCTGGGGAGGTGACAAC
sXbp1	CTGAGTCCGCAGCAGGT	TGAAGAGGCAACAGTGTCAGA
Hspa5	CTGAGGCGTATTTGGGAAAG	CAGCATCTTTGGTTGCTTGTC
Ddit3	CACGTCGATTATATCATGTTGAAGA	GCACTTCCTTCTGGAACACTCT
Atf4	TCAGACACCGGCAAGGAG	TCATCCAACGTGGTCAAGAG
Bax	AGCAAACTGGTGCTCAAGGC	CCACAAAGATGGTCACTGTC
Bcl2	GTGGTGGAGGAACTCTTCAG	GTTCCACAAAGGCATCCCAG

Data S1. Source data and statistical analyses

This Excel file contains the source data used to generate the quantitative graphs in Figs. 1 to 7 and figs. S2 to S8, together with the corresponding statistical analysis results. Worksheets are organized by figure and include the individual values plotted for each panel and statistical outputs, including P values, significance summaries, and test statistics, where applicable.

REFERENCES

1. B. Kyewski, L. Klein, A central role for central tolerance. *Annu. Rev. Immunol.* **24**, 571–606 (2006).

2. S. Inglesfield, E. J. Cosway, W. E. Jenkinson, G. Anderson, Rethinking thymic tolerance: Lessons from mice. *Trends Immunol.* **40**, 279–291 (2019).

3. N. Kadouri, S. Nevo, Y. Goldfarb, J. Abramson, Thymic epithelial cell heterogeneity: TEC by TEC. *Nat. Rev. Immunol.* **20**, 239–253 (2020).

4. T. M. McCaughtry, T. A. Baldwin, M. S. Wilken, K. A. Hogquist, Clonal deletion of thymocytes can occur in the cortex with no involvement of the medulla. *J. Exp. Med.* **205**, 2575–2584 (2008).

5. H. Kurobe, C. Liu, T. Ueno, F. Saito, I. Ohigashi, N. Seach, R. Arakaki, Y. Hayashi, T. Kitagawa, M. Lipp, R. L. Boyd, Y. Takahama, CCR7-dependent cortex-to-medulla migration of positively selected thymocytes is essential for establishing central tolerance. *Immunity* **24**, 165–177 (2006).

6. T. Nitta, S. Nitta, Y. Lei, M. Lipp, Y. Takahama, CCR7-mediated migration of developing thymocytes to the medulla is essential for negative selection to tissue-restricted antigens. *Proc. Natl. Acad. Sci. U.S.A.* **106**, 17129–17133 (2009).

7. E. R. Breed, M. Watanabe, K. A. Hogquist, Measuring thymic clonal deletion at the population level. *J. Immunol.* **202**, 3226–3233 (2019).

8. O. Herbin, A. J. Bonito, S. Jeong, E. G. Weinstein, A. H. Rahman, H. Xiong, M. Merad, K. Alexandropoulos, Medullary thymic epithelial cells and CD8α^{+} dendritic cells coordinately regulate central tolerance but CD8α^{+} cells are dispensable for thymic regulatory T cell production. *J. Autoimmun.* **75**, 141–149 (2016).

9. D. Mathis, C. Benoist, *Aire. Annu. Rev. Immunol.* **27**, 287–312 (2009).

10. J. Březina, M. Vobořil, D. Filipp, Mechanisms of direct and indirect presentation of self-antigens in the thymus. *Front. Immunol.* **13**, 926625 (2022).

11. L. Geng, K. S. L. Lam, A. Xu, The therapeutic potential of FGF21 in metabolic diseases: From bench to clinic. *Nat. Rev. Endocrinol.* **16**, 654–667 (2020).

12. H. Tan, T. Yue, Z. Chen, W. Wu, S. Xu, J. Weng, Targeting FGF21 in cardiovascular and metabolic diseases: From mechanism to medicine. *Int. J. Biol. Sci.* **19**, 66–88 (2023).

13. Y. Nakayama, Y. Masuda, H. Ohta, T. Tanaka, M. Washida, Y.-I. Nabeshima, A. Miyake, N. Itoh, M. Konishi, Fgf21 regulates T-cell development in the neonatal and juvenile thymus. *Sci. Rep.* **7**, 330 (2017).

14. S. A. Wedemeyer, N. E. Jones, I. G. A. Raza, F. M. Green, Y. Xiao, M. K. Semwal, A. K. Garza, K. S. Archuleta, K. L. Wimberly, T. Venables, G. A. Holländer, A. V. Griffith, Paracrine FGF21 dynamically modulates mTOR signaling to regulate thymus function across the lifespan. *Nat. Aging* **5**, 588–606 (2025).

15. Y.-H. Youm, C. Gliniak, Y. Zhang, T. Dlugos, P. E. Scherer, V. D. Dixit, Enhanced paracrine action of FGF21 in stromal cells delays thymic aging. *Nat. Aging* **5**, 576–587 (2025).

16. Y. Hayashi, M. Utsuyama, C. Kurashima, K. Hirokawa, Spontaneous development of organ-specific autoimmune lesions in aged C57BL/6 mice. *Clin. Exp. Immunol.* **78**, 120–126 (1989).

17. W. Jiang, M. S. Anderson, R. Bronson, D. Mathis, C. Benoist, Modifier loci condition autoimmunity provoked by Aire deficiency. *J. Exp. Med.* **202**, 805–815 (2005).

18. Z. Liu, H. Zhang, Y. Hu, D. Liu, L. Li, C. Li, Q. Wang, J. Huo, H. Liu, N. Xie, X. Huang, Y. Liu, C. D. Chen, Y. Shi, X. Zhang, Critical role of histone H3 lysine 27 demethylase Kdm6b in the homeostasis and function of medullary thymic epithelial cells. *Cell Death Differ.* **27**, 2843–2855 (2020).

19. C. S. Constantinescu, N. Farooqi, K. O'Brien, B. Gran, Experimental autoimmune encephalomyelitis (EAE) as a model for multiple sclerosis (MS): EAE as model for MS. *Br. J. Pharmacol.* **164**, 1079–1106 (2011).

20. L. Klein, B. Kyewski, P. M. Allen, K. A. Hogquist, Positive and negative selection of the T cell repertoire: What thymocytes see (and don't see). *Nat. Rev. Immunol.* **14**, 377–391 (2014).

21. N. Thiault, J. Darrigues, V. Adoue, M. Gros, B. Binet, C. Perals, B. Leobon, N. Fazilleau, O. P. Joffre, E. A. Robey, J. P. M. van Meerwijk, P. Romagnoli, Peripheral regulatory T lymphocytes recirculating to the thymus suppress the development of their precursors. *Nat. Immunol.* **16**, 628–634 (2015).

22. D. L. Owen, S. A. Mahmud, L. E. Sjaastad, J. B. Williams, J. A. Spanier, D. R. Simeonov, R. Ruscher, W. Huang, I. Proekt, C. N. Miller, C. Hekim, J. C. Jeschke, P. Aggarwal, U. Broeckel, R. S. LaRue, C. M. Henzler, M.-L. Alegre, M. S. Anderson, A. August, A. Marson, Y. Zheng, C. B. Williams, M. A. Farrar, Thymic regulatory T cells arise via two distinct developmental programs. *Nat. Immunol.* **20**, 195–205 (2019).

23. A. Calindi, L. I. R. Ehrlich, Intrathymic regulation of dendritic cell subsets and their contributions to central tolerance. *Immunol. Rev.* **332**, e70039 (2025).

24. J. S. A. Perry, C.-W. J. Lio, A. L. Kau, K. Nutsch, Z. Yang, J. I. Gordon, K. M. Murphy, C.-S. Hsieh, Distinct contributions of Aire and antigen-presenting-cell subsets to the generation of self-tolerance in the thymus. *Immunity* **41**, 414–426 (2014).

25. N. Itoh, H. Ohta, M. Konishi, Endocrine FGFs: Evolution, physiology, pathophysiology, and pharmacotherapy. *Front. Endocrinol. (Lausanne)* **6**, 154 (2015).

26. C. J. Kroger, N. A. Spidale, B. Wang, R. Tisch, Thymic dendritic cell subsets display distinct efficiencies and mechanisms of intercellular MHC transfer. *J. Immunol.* **198**, 249–256 (2017).

27. V. Millet, P. Naquet, R. R. Guinamard, Intercellular MHC transfer between thymic epithelial and dendritic cells. *Eur. J. Immunol.* **38**, 1257–1263 (2008).

28. J. S. A. Perry, E. V. Russler-Germain, Y. W. Zhou, W. Purtha, M. L. Cooper, J. Choi, M. A. Schroeder, V. Salazar, T. Egawa, B.-C. Lee, N. A. Abumrad, B. S. Kim, M. S. Anderson, J. F. DiPersio, C.-S. Hsieh, Transfer of cell-surface antigens by scavenger receptor CD36 promotes thymic regulatory T cell receptor repertoire development and allo-tolerance. *Immunity* **48**, 923–936.e4 (2018).

29. Y. Hotta, H. Nakamura, M. Konishi, Y. Murata, H. Takagi, S. Matsumura, K. Inoue, T. Fushiki, N. Itoh, Fibroblast growth factor 21 regulates lipolysis in white adipose tissue but is not required for ketogenesis and triglyceride clearance in liver. *Endocrinology* **150**, 4625–4633 (2009).

30. E. K. Schmidt, G. Clavarino, M. Ceppi, P. Pierre, SUnSET, a nonradioactive method to monitor protein synthesis. *Nat. Methods* **6**, 275–277 (2009).

31. C. St-Pierre, E. Morgand, M. Benhammadi, A. Rouette, M.-P. Hardy, L. Gaboury, C. Perreault, Immunoproteasomes control the homeostasis of medullary thymic epithelial cells by alleviating proteotoxic stress. *Cell Rep.* **21**, 2558–2570 (2017).

32. T. Liu, S. Xia, The proteostasis of thymic stromal cells in health and diseases. *Protein J.* **43**, 447–463 (2024).

33. S. Jiang, C. Yan, Q.-C. Fang, M.-L. Shao, Y.-L. Zhang, Y. Liu, Y.-P. Deng, B. Shan, J.-Q. Liu, H.-T. Li, L. Yang, J. Zhou, Z. Dai, Y. Liu, W.-P. Jia, Fibroblast growth factor 21 is regulated by the IRE1α-XBP1 branch of the unfolded protein response and counteracts endoplasmic reticulum stress-induced hepatic steatosis. *J. Biol. Chem.* **289**, 29751–29765 (2014).

34. S. H. Kim, K. H. Kim, H.-K. Kim, M.-J. Kim, S. H. Back, M. Konishi, N. Itoh, M.-S. Lee, Fibroblast growth factor 21 participates in adaptation to endoplasmic reticulum stress and attenuates obesity-induced hepatic metabolic stress. *Diabetologia* **58**, 809–818 (2015).

35. K. Pakos-Zebrucka, I. Koryga, K. Mnich, M. Ljujic, A. Samali, A. M. Gorman, The integrated stress response. *EMBO Rep.* **17**, 1374–1395 (2016).

36. P. Liang, L. Zhong, L. Gong, J. Wang, Y. Zhu, W. Liu, J. Yang, Fibroblast growth factor 21 protects rat cardiomyocytes from endoplasmic reticulum stress by promoting the fibroblast

growth factor receptor 1-extracellular signal-regulated kinase 1/2 signaling pathway. *Int. J. Mol. Med.* **40**, 1477–1485 (2017).

37. S. N. Sansom, N. Shikama-Dorn, S. Zhanybekova, G. Nusspaumer, I. C. Macaulay, M. E. Deadman, A. Heger, C. P. Ponting, G. A. Holländer, Population and single-cell genomics reveal the Aire dependency, relief from Polycomb silencing, and distribution of self-antigen expression in thymic epithelia. *Genome Res.* **24**, 1918–1931 (2014).

38. M. Costa-Mattioli, P. Walter, The integrated stress response: From mechanism to disease. *Science* **368**, eaat5314 (2020).

39. X. Dong, Z. Liang, J. Zhang, Q. Zhang, Y. Xu, Z. Zhang, L. Zhang, B. Zhang, Y. Zhao, Trappc1 deficiency impairs thymic epithelial cell development by breaking endoplasmic reticulum homeostasis. *Eur. J. Immunol.* **52**, 1789–1804 (2022).

40. N. Yamaguchi, Y. Takakura, T. Akiyama, Autophagy and proteasomes in thymic epithelial cells: Essential bulk protein degradation systems for immune homeostasis maintenance. *Front. Immunol.* **15**, 1488020 (2024).

41. J. Derbinski, S. Pinto, S. Rösch, K. Hexel, B. Kyewski, Promiscuous gene expression patterns in single medullary thymic epithelial cells argue for a stochastic mechanism. *Proc. Natl. Acad. Sci. U.S.A.* **105**, 657–662 (2008).

42. M. Vobořil, J. Březina, T. Brabec, J. Dobeš, O. Ballek, M. Dobešová, J. Manning, R. S. Blumberg, D. Filipp, A model of preferential pairing between epithelial and dendritic cells in thymic antigen transfer. *eLife* **11**, e71578 (2022).

43. T. Venables, A. V. Griffith, A. DeAraujo, H. T. Petrie, Dynamic changes in epithelial cell morphology control thymic organ size during atrophy and regeneration. *Nat. Commun.* **10**, 4402 (2019).

44. A. V. Griffith, M. Fallahi, H. Nakase, M. Gosink, B. Young, H. T. Petrie, Spatial mapping of thymic stromal microenvironments reveals unique features influencing T lymphoid differentiation. *Immunity* **31**, 999–1009 (2009).

45. M. Kasai, K. Hirokawa, A novel cofactor produced by a thymic epithelial cell line: Promotion of proliferation of immature thymic lymphocytes by the presence of interleukin-1 and various mitogens. *Cell. Immunol.* **132**, 377–390 (1991).

46. T. Mizuochi, M. Kasai, T. Kokuho, T. Kakiuchi, K. Hirokawa, Medullary but not cortical thymic epithelial cells present soluble antigens to helper T cells. *J. Exp. Med.* **175**, 1601–1605 (1992).

47. M. Kasai, T. Mizuochi, Derivation, culture, and characterization of thymic epithelial cell lines. *Methods Mol. Biol.* **380**, 107–123 (2007).

48. A. M. Bolger, M. Lohse, B. Usadel, Trimmomatic: A flexible trimmer for Illumina sequence data. *Bioinformatics* **30**, 2114–2120 (2014).

49. D. Kim, B. Langmead, S. L. Salzberg, HISAT: A fast spliced aligner with low memory requirements. *Nat. Methods* **12**, 357–360 (2015).

50. M. Pertea, G. M. Pertea, C. M. Antonescu, T. C. Chang, J. T. Mendell, S. L. Salzberg, StringTie enables improved reconstruction of a transcriptome from RNA-seq reads. *Nat. Biotechnol.* **33**, 290–295 (2015).

51. M. I. Love, W. Huber, S. Anders, Moderated estimation of fold change and dispersion for RNA-seq data with DESeq2. *Genome Biol.* **15**, 550 (2014).

52. A. Subramanian, P. Tamayo, V. K. Mootha, S. Mukherjee, B. L. Ebert, M. A. Gillette, A. Paulovich, S. L. Pomeroy, T. R. Golub, E. S. Lander, J. P. Mesirov, Gene set enrichment analysis: A knowledge-based approach for interpreting genome-wide expression profiles. *Proc. Natl. Acad. Sci. U.S.A.* **102**, 15545–15550 (2005).

53. I. M. Stromnes, J. M. Goverman, Active induction of experimental allergic encephalomyelitis. *Nat. Protoc.* **1**, 1810–1819 (2006).
